# Investigation of Clinically Significant Molecular Aberrations in Patients with Prostate Cancer: Implications for Personalized Treatment, Prognosis and Genetic Testing

**DOI:** 10.3390/ijms241411834

**Published:** 2023-07-23

**Authors:** Elena Fountzilas, Maria Kouspou, Alexia Eliades, Kyriaki Papadopoulou, Evangelos Bournakis, Anna Goussia, Marinos Tsiatas, Achilleas Achilleos, Kyriakos Tsangaras, Gaetan Billioud, Charalambos Loizides, Christos Lemesios, Elena Kypri, Marios Ioannides, George Koumbaris, Sofia Levva, Ioannis Vakalopoulos, Athanasios Paliouras, Stavroula Pervana, Filippos Koinis, Redi Bumci, Athina Christopoulou, Soultana Meditskou, Amanda Psyrri, Ioannis Boukovinas, Anastasios Visvikis, Vasilios Karavasilis, George K. Koukoulis, Athanasios Kotsakis, Dimitrios Giannakis, George Fountzilas, Philippos C. Patsalis

**Affiliations:** 1 Department of Medical Oncology, St. Lukes’s Hospital, 55236 Thessaloniki, Greece; 2 Medical Oncology, German Oncology Center, European University Cyprus, Limassol 3036, Cyprus; 3 Department of Basic and Clinical Sciences, University of Nicosia Medical School, Nicosia 2417, Cyprus; kouspou.ma@live.unic.ac.cy (M.K.); philippos.patsalis@medicover.com (P.C.P.); 4 Medicover Genetics, Nicosia 2409, Cyprus; alexia.eliades@medicover.com (A.E.); achilleas.achilleos@medicover.com (A.A.); kyriakos.tsangaras@medicover.com (K.T.); gaetan.billioud@medicover.com (G.B.); charalambos.loizides@medicover.com (C.L.); christos.lemesios@medicover.com (C.L.); elena.kypri@medicover.com (E.K.); marios.ioannides@medicover.com (M.I.); george.koumbaris@medicover.com (G.K.); 5 Laboratory of Molecular Oncology, Hellenic Foundation for Cancer Research/Aristotle University of Thessaloniki, 54124 Thessaloniki, Greece; kyriakipapadopoulou@hotmail.com (K.P.); fountzil@auth.gr (G.F.); 6 Oncology Unit, 2nd Department of Surgery, Aretaieio Hospital, Medical School, National and Kapodistrian University of Athens, 11528 Athens, Greece; vagimith@yahoo.com; 7 Oncologic Clinical Trials and Research Clinic, Metropolitan General Hospital, 15562 Athens, Greece; 8 Department of Pathology, Faculty of Medicine, Ioannina University Hospital, 45500 Ioannina, Greece; goussiaanna@gmail.com (A.G.); redibumci@gmail.com (R.B.); 9 Department of Pathology, German Oncology Center, Limassol 4108, Cyprus; 10 Department of Oncology, Athens Medical Center, 15125 Marousi, Greece; mtsiatas@otenet.gr; 11 Medical Oncology, Bioclinic of Thessaloniki, 54622 Thessaloniki, Greece; dr.slevva@gmail.com (S.L.); ibouk@otenet.gr (I.B.); 12 First Department of Urology, School of Medicine, Aristotle University of Thessaloniki, “G. Gennimatas” General Hospital, 54124 Thessaloniki, Greece; vakalj@otenet.gr; 13 Department of Urology, Ioannina University Hospital, 45500 Ioannina, Greece; paliouras-a@hotmail.com; 14 Department of Pathology, Papageorgiou Hospital, 56429 Thessaloniki, Greece; st.pervana@gmail.com; 15 Department of Medical Oncology, University General Hospital of Larissa, 41110 Larissa, Greece; phillipkoinis@gmail.com (F.K.); thankotsakis@hotmail.com (A.K.); 16 Medical Oncology Unit, S. Andrew Hospital, 26332 Patras, Greece; athinachristo@hotmail.com; 17 Department of Histology-Embryology, School of Medicine, Aristotle University of Thessaloniki, 54124 Thessaloniki, Greece; meditskou@gmail.com; 18 Section of Medical Oncology, Department of Internal Medicine, Attikon University Hospital, Faculty of Medicine, National and Kapodistrian University of Athens School of Medicine, 12462 Athens, Greece; psyrri237@yahoo.com; 19 Third Department of Medical Oncology, Agii Anargiri Cancer Hospital, 14564 Athens, Greece; anvisvikis@gmail.com; 20 University College London Hospitals NHS Foundation Trust, London WC1N 3BG, UK; karavasv@auth.gr; 21 Department of Pathology, Faculty of Medicine, School of Health Sciences, University of Thessaly, 41100 Larissa, Greece; gkgk@otenet.gr; 22 Department of Urology, Faculty of Medicine, University of Ioannina, 45110 Ioannina, Greece; dgiannak@cc.uoi.gr; 23 Aristotle University of Thessaloniki, 54124 Thessaloniki, Greece; 24 Department of Medical Oncology, German Oncology Center, Limassol 4108, Cyprus

**Keywords:** prostate cancer, molecular profiling, genomics, mutations, TMPRSS2-ERG, BRCA2, overall survival, prognostic

## Abstract

The data on tumor molecular profiling of European patients with prostate cancer is limited. Our aim was to evaluate the prevalence and prognostic and predictive values of gene alterations in unselected patients with prostate cancer. The presence of gene alterations was assessed in patients with histologically confirmed prostate cancer using the ForeSENTIA^®^ Prostate panel (Medicover Genetics), targeting 36 clinically relevant genes and microsatellite instability testing. The primary endpoint was the prevalence of gene alterations in homologous recombination repair (HRR) genes. Overall, 196 patients with prostate cancer were evaluated (median age 72.2 years, metastatic disease in 141 (71.9%) patients). Gene alterations were identified in 120 (61%) patients, while alteration in HRR genes were identified in 34 (17.3%) patients. The most commonly mutated HRR genes were *ATM* (17, 8.7%), *BRCA2* (9, 4.6%) and *BRCA1* (4, 2%). The presence of HRR gene alterations was not associated with advanced stage (*p* = 0.21), age at diagnosis (*p* = 0.28), Gleason score (*p* = 0.17) or overall survival (HR 0.72; 95% CI: 0.41–1.26; *p* = 0.251). We identified clinically relevant somatic gene alterations in European patients with prostate cancer. These molecular alterations have prognostic significance and therapeutic implications and/or may trigger genetic testing in selected patients. In the era of precision medicine, prospective research on the predictive role of these alterations for innovative treatments or their combinations is warranted.

## 1. Introduction

Prostate cancer is the most frequent cancer in men. Recent studies have reported clinically significant germline pathogenic variants (PVs) in DNA repair genes, mainly those participating in the homologous recombination pathway, including PVs in *BRCA1, BRCA2, ATM* and *CHEK2*. In addition, germline mutations in mismatch repair (MMR) genes have also been identified in a smaller proportion of patients with prostate cancer [1,2]. Finally, PVs in *HOXB13*, a tumor suppressor gene, have been implicated in increasing the predisposition for prostate cancer [3]. Somatic mutations in DNA repair genes have also been reported in prostate tumor tissue [4]. 

Importantly, PVs in different genes have been associated with poor prognosis in patients with prostate cancer. Patients with localized prostate cancer and carrying *BRCA2* germline mutations have poorer clinical outcomes compared to patients without mutations [5,6,7]. Studies have shown that *BRCA2*-associated prostate tumors exhibit an aggressive phenotype and are often associated with the presence of the intraductal carcinoma of the prostate pathology, a poor prognostic feature for prostate cancer [8].

The identification of germline and somatic mutations has several therapeutic implications. Mutations in genes participating in the homologous recombination repair (HRR) system have been shown to be associated with clinical benefit from poly-ADP ribose polymerase (PARP) inhibitors [4,9,10,11,12]. Initially, olaparib was evaluated in a phase II clinical trial in patients with metastatic castration-resistant prostate cancer (mCRPC) [4]. Among the 16 patients with homozygous deletions and/or deleterious mutations in DNA-repair genes (*BRCA1/2*, *ATM*, *CHEK2* and Fanconi’s anemia genes), 14 (88%) had a response to treatment with olaparib. A randomized, double-blind, placebo-controlled, phase 2 trial evaluated the combination of olaparib with abiraterone in patients with mCRPC and demonstrated that the combination led to increased radiographic progression-free survival compared to abiraterone monotherapy [13]. The TRITON2 was a phase II trial that evaluated the administration of rucaparib to patients with metastatic mCRPC associated with a *BRCA* alteration [11]. The objective response rate was 43.5% in patients with measurable disease. Recently, rucaparib was shown to be associated with longer imaging-based progression-free survival (PFS) compared to the physician’s choice in patients with metastatic, castration-resistant prostate cancer with a BRCA alteration [14]. In another phase II trial (TALAPRO-1), the benefit of talazoparib was evaluated in previously treated patients with mCRPC and PVs in HRR genes [12]. The objective response rate was 29.8% in the patients of the study. Based on the promising results of the aforementioned clinical trials, olaparib has been approved by the Food and Drug Administration (FDA) for patients with mCRPC and deleterious or suspected deleterious germline or somatic mutations in HRR genes, who have progressed following prior treatment with enzalutamide or abiraterone. Meanwhile, rucaparib has been approved for the treatment of patients with mCRPC and deleterious *BRCA* mutations who have progressed after prior treatment with androgen receptor-directed therapy and a taxane-based chemotherapy. Finally, immune checkpoint inhibitors have shown significant clinical efficacy in patients with microsatellite instability (MSI)-high prostate cancer [1]. 

Meanwhile, a significant number of investigators have evaluated the prevalence of germline PVs in patients with prostate cancer, data regarding the prevalence of somatic PVs is limited. Our aim was to evaluate the prevalence of clinically relevant somatic PVs in patients with metastatic, locally advanced or high-grade prostate cancer and assess their prognostic and predictive role in those patients. 

## 2. Results

### 2.1. Patient Characteristics

Overall, 219 patients with prostate cancer were evaluated in this study. Twenty-three patients were excluded due to insufficient or low-quality DNA extracted from the FFPE specimen. The median age at diagnosis was 72.2 years (IQR: 64.3, 75.2). The majority of patients (141 patients, 71.9%) presented with metastatic disease. Of the 184 patients with available data for family history, 36 (19.6%) reported a family history of cancer, while 10 (5.4%) had a history of prostate cancer, specifically. The patients’ detailed clinicopathological characteristics are summarized in Table 1.

### 2.2. Tumor Molecular Profiling

**Figure 1:**
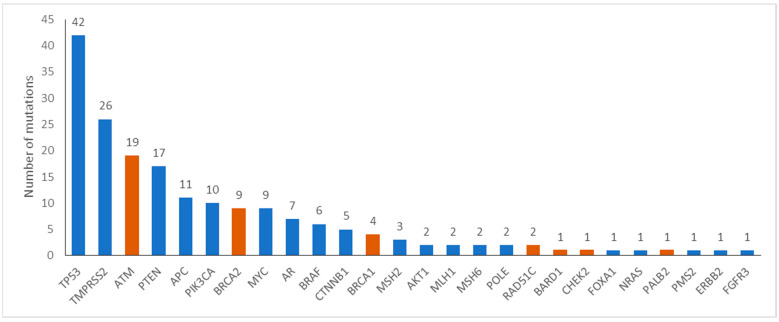
Number of patients with mutations in the respective genes. Orange color denotes homologous recombination repair genes.

Tumor molecular alterations were identified in 120 of 196 (61%) patients. The most commonly mutated genes are shown in Figure 1. Specifically, alterations in HRR genes were reported in 34 (17.3%) patients, in 14.3% and 3.1% of patients with metastatic and early disease, respectively. Commonly mutated HRR genes were *ATM* (17, 8.7%), *BRCA2* (9, 4.6%) and *BRCA1* (4, 2%). Rearrangements in *TMPRSS2-ERG*, a potentially prognostic gene, were identified in 26 (13.3%) patients. Eight (4%) patients harbored alterations in MMR genes. 

**Figure 2:**
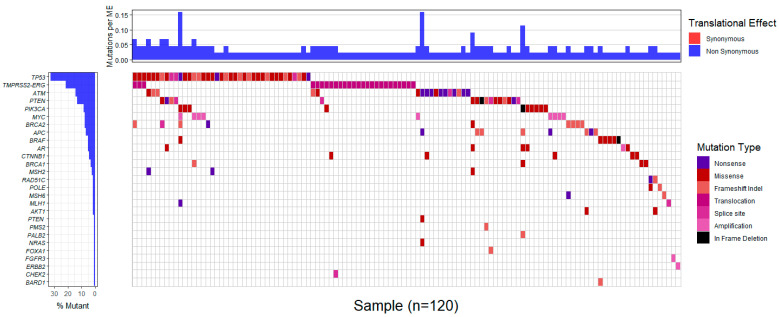
Map showing profiled gene mutations among 120 tumors bearing tumor molecular alterations.

Markedly, comparison of the genotypes of 26 patients’ tumors sequenced with both the Medicover Genetic’s prostate cancer assay and the custom Ampliseq IAD207308_231 panel yielded similar results in all but four instances. Specifically, tumor profiling with both panels ascertained the lack of clinically significant variants in 9 patients and the presence of 12 alterations in *ATM*, *BRCA2*, *CTNNB1*, *MSH2*, *PIK3CA*, *PTEN* and *TP53* in 11 patients. Meanwhile, *TMPRSS2-ERG* rearrangements and *AR* or *MYC* amplifications, present in the tumors of 6 out of the aforementioned 26 patients, were exclusively identified by the Medicover Genetic’s prostate cancer assay, since such structural variants are not targeted by the Ampliseq IAD207308_231 panel. Of note, four variants were retrieved only with the Medicover Genetic’s prostate cancer assay in three patient tumors; these variants were targeted by the Ampliseq panel as well, which, considering the respective samples’ good quality metrics in either case, could reflect the tumor’s heterogeneity in terms of different subclones. In addition, microsatellite instability was detected in 6 out of 196 patients (3.1%) (Supplemental Table S3). The profiles of gene alterations in the affected tumors are shown in Figure 2 and Supplemental Table S3.

Sanger sequencing for germline analysis was implemented in five patients with an available peripheral blood sample, and the PVs that were identified in the tumor implicated a germline origin. In all five patients, a germline status was confirmed in the following HRR genes: *RAD51C* (one patient) and *ATM* (four patients) (Supplemental Figure S1).

### 2.3. Clinical Associations

A family history of cancer was more commonly reported in patients with tumors harboring mutations in HRR genes (11 patients, 35.5%) compared to the rest of the patients (25, 16.3%) (chi-square *p* = 0.014). The presence of HRR gene alterations (either of somatic or of germline origin) was not associated with advanced stage (*p* = 0.21), age at diagnosis (*p* = 0.28) or Gleason score (*p* = 0.17).

#### Clinical Outcomes

**Figure 3:**
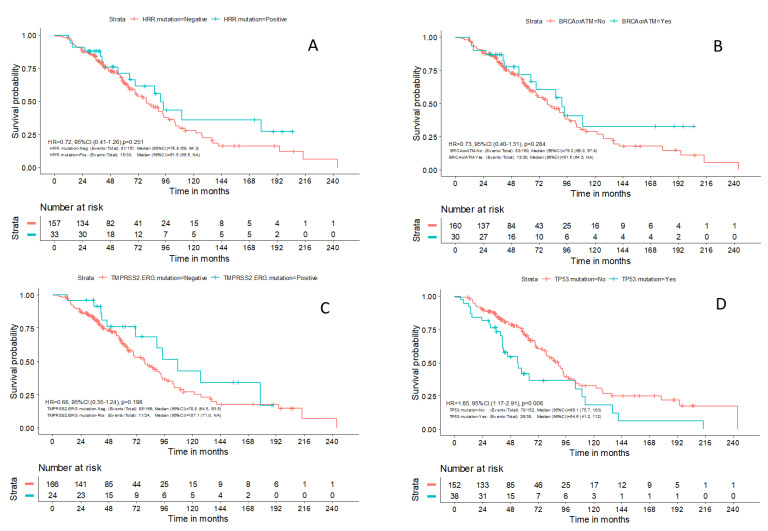
Prognostic significance of mutations in our patient cohort. There was no difference in overall survival (OS) between patients with and without mutations in (**A**) homologous recombination repair genes (HR 0.72; 95% CI: 0.41–1.26; *p* = 0.251), (**B**) *BRCA1/2* and *ATM* genes (HR 0.73; 95% CI: 0.40–1.31; *p* = 0.284) and (**C**) *TMPRSS2* rearrangements (HR 0.66; 95% CI: 0.35–1.24; *p* = 0.198). (**D**) The presence of *TP53* mutations was associated with worse overall survival in patients with prostate cancer (HR 1.85; 95% CI: 1.17–2.91; *p* = 0.008).

Within a median follow-up of 49.5 months (0.1–243.5), a total of 96 (49%) deaths were reported. The median OS for patients with available outcome data (N = 190) was 81 months (95% CI: 69.5–94.3). There was no difference in OS between patients with advanced prostate cancer and PVs in HRR genes compared to patients without PVs in those genes (HR 0.72; 95% CI: 0.41–1.26; *p* = 0.251) or in patients with *BRCA1/2* and *ATM* mutations compared to the rest of the patients (HR 0.73; 95% CI: 0.40–1.31; *p* = 0.284) (Figure 3A and Figure 3B, respectively). Despite previously reported data, mutations in *TMPRSS2-ERG* were not associated with prognosis in the patients of our study (HR 0.66; 95% CI: 0.35–1.24; *p* = 0.198) (Figure 3C). The patients with mutations in *TP53* had a worse OS compared to the rest of the patients [54.6 months (95%CI 41.2–112.0) vs. 89.1 months (95% CI 75.7–103.0), HR 1.85; 95% CI: 1.17–2.91; *p* = 0.008] (Figure 3D). Finally, no association was identified in terms of PFS between the aforementioned molecular subgroups of patients. Only one patient with HRR alteration was treated with olaparib; therefore, no association with response to PARP inhibitors could be performed.

## 3. Discussion

Tumor molecular profiling is being extensively used to assess for the presence of clinically relevant PVs that will enable individualization of treatment of patients with cancer. However, tumor molecular data in patients with prostate cancer associated with clinical data are scarce. In this study, we evaluated the prevalence of somatic PVs in clinically relevant genes and combined clinical and genomic data to investigate their prognostic or predictive significance in European patients with prostate cancer. We indeed identified clinically relevant pathogenic variants in the patients of our study and demonstrated the prognostic potential for select genes. These molecular alterations have prognostic and therapeutic implications and/or may trigger genetic testing in selected patients. 

The commonly mutated genes included *TP53, TMPRSS2-ERG, ATM* and *PTEN*. The majority of *TP53* variants laid within exons 4–8 which encode for the DNA binding domain of the T53 protein and is a hotspot region frequently mutated across multiple cancer types [15]. Likewise, most *APC* mutations were frameshift and nonsense mutations that led to an early termination of the amino acid sequence and resulted in a loss of the FAT and/or PI3K/PI4K catalytic domain and thereby a loss of ATM protein function [16]. In addition, *PTEN* mutations in hotspot regions in the P-loop motif (p.Arg130Ter) as well as in the greater phosphatase domain (p.Arg173His, p.Gly132Asp, p.Pro169_Ser170insIle, p.Ala34CysfsTer10, p.Tyr27Cys) were identified in our cohort. The frequencies of these mutated genes were similar to those of previously reported data in publicly available cancer somatic databases such as COSMIC and TCGA [17,18]. We did not identify any differences in OS or PFS between patients with molecular alterations in HRR genes and the rest of the patients. Importantly, patients with PVs in *TP53* had a worse OS compared to the rest of the patients, underlying the need for improvement of management of these patients. 

While several investigators have evaluated the prevalence and clinical significance of PVs in patients with prostate cancer, they mostly focused on germline PVs [19]. One of the largest studies evaluated 1302 tumors from patients with prostate cancer, who had undergone next-generation sequencing using FoundationOne or FoundationOne CDx assays [20]. This study reported on the prevalence of tumor molecular alterations in these patients, and also demonstrated the stability of molecular alterations in HRR genes during tumor progression. No clinical outcomes were reported in this study; therefore, the prognostic or predictive significance of these alterations could not be addressed [20]. Another study also demonstrated limited genomic evolution in patients with lung-recurrent hormone-sensitive prostate cancer [21]. Finally, similarly to our study, genomic analysis of 185 tumors demonstrated PVs in DNA damage repair genes in 19% of patients. The presence of DNA damage repair genes was associated with high-volume disease [22]. 

In our study, the presence of PVs in HRR genes was not associated with OS in patients with advanced prostate cancer. Similarly, no association was identified in the PROREPAIR-B, a prospective study evaluating clinical outcomes depending on germline PVs in patients with metastatic castration-resistant prostate cancer. The investigators showed no difference in cause-specific survival between *ATM*/*BRCA1*/*BRCA2*/*PALB2* carriers and noncarriers. The study did, however, report that patients with metastatic castration-resistant prostate cancer and germline *BRCA2* PVs had a worse OS compared to patients without such PVs. Other investigators demonstrated that germline PVs in *BRCA1, BRCA2, PALB2* or *ATM* were independently associated with a shorter OS [23]. However, disease prognosis in patients with PVs may improve with the inclusion of targeted agents, including PARP inhibitors and immunotherapy, in the treatment of patients with advanced prostate cancer. 

Importantly, the predictive role of PVs in HRR genes needs to be evaluated further. Previous studies suggest that the presence of PVs in HRR genes is associated with clinical benefit from diverse treatments. For instance, one study showed that patients with BRCA2 germline PVs had improved cause-specific survival and second PFS when abiraterone or enzalutamide was administered as the first-line treatment compared to taxane therapy [24]. Other investigators demonstrated that patients with *BRCA2* PVs had a significantly longer median PFS with PARP inhibitors compared to those with molecular alterations in other HRR genes [25]. Finally, germline PVs in *BRCA1*, *BRCA2*, *PALB2* or *ATM* were independently associated with short time to castration in patients with advanced prostate cancer [23]. This analysis was precluded in our dataset due to the lack of statistical power.

The limitations of our study include the retrospective sample collection, the inclusion of patients with heterogenous disease stages and the limited number of patients treated with PARP inhibitors and/or platinum agents. In addition, germline testing was performed in select patients. The strengths of the study include the large number of patients with prostate cancer who underwent tumor molecular profiling and the association with clinical outcomes. 

In conclusion, tumor molecular profiling demonstrated clinically significant PVs in patients with prostate cancer. In our study, PVs in HRR genes were not associated with OS in patients with advanced cancer. On the contrary, patients with PVs in *TP53* had a worse OS compared to the rest of the patients. As innovative agents and their combinations are being approved for the treatment of patients with prostate cancer, the evaluation of the predictive role of tumor molecular alterations for clinical benefit from these agents is warranted.

## 4. Materials and Methods

### 4.1. Patients

Our study included patients with recurrent, locally advanced, metastatic and/or high-grade operable prostate cancer. Formalin-fixed, paraffin-embedded, tumor tissue was retrieved from Pathology Laboratories, accompanied by peripheral blood samples obtained from patients, when possible. The samples were retrospectively and prospectively collected from patients who received treatment at Hellenic Cooperative Oncology Group (HeCOG)-affiliated departments of oncology through the years 1995–2019. Clinicopathologic characteristics were retrieved from patient medical records. Histological subtype and Gleason score were recorded from the pathology reports. Importantly, a detailed family history was recorded, when available. The data collection was conducted in compliance with the regulations of the bioethics committees of the participating hospitals. The study was approved by the Institutional Review Boards of General Hospital “Agioi Anargiri” (17721/29.10.2019), “Euroclinic” (117/28.5.2019), University Hospital of Larisa (16/8/11/6/20) and the Cyprus National Bioethics Committee (EEBK/EP/2021/18).

### 4.2. Sample Evaluation

Available FFPE tumor blocks were subjected to histological review by an experienced pathologist to evaluate H&E sections to confirm the diagnosis, histologic type, grade and tumor cell content (TCC%); tumor dense areas were also marked for manual macrodissection, prior to DNA extraction, in order to enrich samples for tumor DNA. Macrodissection was performed at the Laboratory of Molecular Oncology (LMO), Hellenic Foundation for Cancer Research (HeFCR)/Aristotle University of Thessaloniki. TCC% was measured as tumor nuclei vs. all nuclei in the areas marked for macrodissection. Tumor DNA extraction was performed from approximately five to ten 10 μm FFPE whole sections, following manual macrodissection, using the QIAGEN GeneRead DNA FFPE (Qiagen, Hilden, Germany) kit according to standard procedures. A Qubit dsDNA High Sensitivity (HS) kit was used with a Qubit 3.0 Fluorometer (Invitrogen, Thermo Fisher Scientific, Waltham, MA, USA) to quantify the extracted dsDNA of the FFPE samples. Moreover, for assessment of germline status, germline DNA was also extracted from peripheral blood samples from 5 patients (QIamp DNA Blood Midi Kit, Qiagen, Hilden, Germany) according to manufacturer’s instructions.

### 4.3. Library Preparation, Target Capture Enrichment and Next-Generation Sequencing

The sample preparation for NGS was performed as described previously [26]. Briefly, a Lotus DNA Library Prep Kit (Integrated DNA Technologies, Clareville, Iowa) was used to prepare the DNA libraries from the extracted DNA samples according to manufacturer’s instructions. Briefly, 60–250 ng of DNA were subjected to enzymatic fragmentation at 32 °C for 7 min, followed by adaptor ligation at 20 °C for 20 min and clean up using magnetic beads. Next, the samples were further subjected to indexing PCR and final bead-based clean up. DNA concentration was measured through the Qubit fluorometric method and evaluation of the DNA libraries was performed through the 4150 Agilent Tapestation System (D100 ScreenTape and High Sensitivity D1000 ScreenTape Agilent, Santa Clara, CA, USA). Enrichment via hybridization capture-based NGS assay was utilized to capture target sequences from the DNA library samples. This technology was based on the hybridization of regions of interest to biotinylated probes called Target Capture Sequences (TACS) that were specifically designed to target selected genomic loci. The biotinylated probes were immobilized on streptavidin residues on magnetic beads and the DNA libraries were then hybridized. A list of the 36 genes targeted by Medicover Genetic’s (Berlin, Germany) prostate cancer assay is shown in Supplemental Table S1. Enriched libraries were then subjected to NGS using Illumina platforms.

Furthermore, for the purpose of evaluating the performance of other NGS platforms and panels on the detection of somatic SNVs and indels, a small subset of the patients’ tumor samples, selected at random, was also sequenced at LMO’s Ion Proton Platform, using a targeted, custom, Ampliseq panel (IAD207308_231) (Supplemental Table S2) that shares common targets with the Medicover Genetic’s prostate cancer assay, primarily DNA damage repair genes and others with clinical relevance in prostate cancer [27,28,29]. Library preparation with an Ampliseq Library Kit v.2.0 (Life Technologies, Carlsbad, CA, USA) and Ampliseq primers was performed with standard protocols, as published previously [30]. The resulting libraries were clonally amplified using the One-Touch-2 instrument and enriched using the OneTouch ES with the Ion PI Hi-Q OT2 200 Kit, followed by Ion Proton sequencing with an Ion PI Hi-Q Sequencing 200 Kit (Life Technologies, Carlsbad, CA, USA). Data retrieval and base calling were then accomplished on the Torrent Server (v5.12.0.4) with variant allele frequencies (VAFs) of >5% accepted by default. Concerning the NGS metrics of tumor samples sequenced on the Ion Proton, the median mean depth was 3587 (mean: 3748; min–max: 1141–12928) and the median number of amplicons with ≥100 reads was 99.0% (mean: 98.44%; min–max: 87.64–99.48%).

### 4.4. Bioinformatics Analysis

Bioinformatic tools were applied for demultiplexing of the NGS output files (bcl2fastq (v.2.16.0) and alignment of reads to the GRCh37/hg19 human reference genome using the Burrows–Wheeler alignment algorithm [31]. Duplicate read entries were removed and aligned reads files were converted to a binary (BAM) format [32]. Variant calling was performed using a sensitive and versatile variant caller [33]. The annotation of variant calls was carried out by VEP [34]. Variant classification was performed according to AMP/ASCO/CAP guidelines using two variant interpretation platforms, CGI and Varsome [35,36,37]. Variants of strong clinical significance (Tier 1) and potential clinical significance (Tier2) were used for data analysis [38]. Copy number alterations (CNAs) were detected using a circular binary segmentation algorithm. Gene rearrangements were identified using structural variant calling algorithms based on discordant pair and split-read information or local assembly [39,40,41] followed by an in-house filtering pipeline. One hundred microsatellite loci (repetitive DNA sequences) across the genome were targeted for the assessment of microsatellite instability. Instability was assessed for each targeted microsatellite region. An adjusted cumulative score was generated representing the fraction of unstable loci and considering only the MSI loci that achieve sufficient coverage for each sample, based on a linear regression model trained on samples with known MSI status (using a PCR-based method). Samples with an MSI score of at least 20 were classified as microsatellite high.

### 4.5. Sanger Sequencing 

Sanger sequencing was performed for 5 patients with suspected germline alterations identified in their tumor sample. DNA was extracted from peripheral blood as described above and subjected to sequencing using an ABI 3130xl Genetic Analyzer (Thermo Fisher Scientific, Waltham, MA, USA).

### 4.6. Statistical Analysis

Numeric variables are summarized using the median alongside the minimum and maximum values. Categorical variables are presented using frequencies and percentages. Associations between categorical variables were tested using the chi-squared test while the Mann–Whitney test was used to examine associations between categorical and continuous variables. The significance level was set at a two-sided 0.05. Overall survival (OS) was defined as the time from date of initial diagnosis until the date of death (from any cause) or last contact. PFS was assessed in patients with advanced cancer and was defined as the time from first-line treatment initiation until disease progression, death (from any cause) or last contact. The Kaplan–Meier method was used to calculate the OS and PFS probability since diagnosis and chemotherapy initiation, respectively, for the patient subgroups. Hazard ratios generated by the Cox regression model and median survival were presented alongside 95% confidence interval (C.I.) The log-rank test was used to examine whether there was a statistically significant difference between survival functions. Statistical analyses were performed using the SAS software (SAS version 9.4, SAS Institute Inc. Cary, NC, USA) and R language (R Core Team: R: A Language and Environment for Statistical Computing Vienna, Austria: Foundation for Statistical Computing. Available from: http://www.R-project.org/ (accessed on 20 May 2023), R version 4.2.2, 2022-10-31 ucrt). Ggplot2 R package, survminer R package and survival packages were employed to conduct the survival analysis and present the Kaplan–Meier curves. A map showing the profiled gene mutations was generated using BiocManager and GenVisR R packages.

## Figures and Tables

**Table 1:** Patient characteristics.

Factor	Total (N = 196)
Age at diagnosis	69.6 (46.8, 95.0)
PSA at diagnosis *	28.0 (0.01, 4155.0)
Family history of cancer *	
No	148 (80.4)
Yes	36 (19.6)
Family history of prostate cancer *	
No	176 (94.6)
Yes	10 (5.4)
Risk group *	
Low	9 (6.8)
Intermediate	19 (14.3)
High	40 (30.1)
Very high	64 (48.1)
Unknown	1 (0.75)
Gleason Score *	
2–6	15 (7.9)
7	40 (21.0)
8–9	125 (65.8)
10	10 (5.3)
Metastasis	
No	55 (28.1)
Yes	141 (71.9)

* Data not available for all subjects. Missing values: age at diagnosis = 1, PSA at diagnosis = 51, family history of cancer = 12, family history of prostate cancer = 10, risk group = 63, Gleason score = 6. Values presented as median (min, max) or N (column %).

## Data Availability

Data available upon request.

## Supplementary Materials

The supporting information can be downloaded at: https://www.mdpi.com/article/10.3390/ijms241411834/s1.

## References

[1] Abida W., Cheng M.L., Armenia J., Middha S., Autio K.A., Vargas H.A., Rathkopf D., Morris M.J., Danila D.C., Slovin S.F. (2019). Analysis of the Prevalence of Microsatellite Instability in Prostate Cancer and Response to Immune Checkpoint Blockade. JAMA Oncol..

[2] Nicolosi P., Ledet E., Yang S., Michalski S., Freschi B., O’Leary E., Esplin E.D., Nussbaum R.L., Sartor O. (2019). Prevalence of Germline Variants in Prostate Cancer and Implications for Current Genetic Testing Guidelines. JAMA Oncol..

[3] Ewing C.M., Ray A.M., Lange E.M., Zuhlke K.A., Robbins C.M., Tembe W.D., Wiley K.E., Isaacs S.D., Johng D., Wang Y. (2012). Germline mutations in HOXB13 and prostate-cancer risk. N. Engl. J. Med..

[4] Mateo J., Carreira S., Sandhu S., Miranda S., Mossop H., Perez-Lopez R., Nava Rodrigues D., Robinson D., Omlin A., Tunariu N. (2015). DNA-Repair Defects and Olaparib in Metastatic Prostate Cancer. N. Engl. J. Med..

[5] Castro E., Goh C., Olmos D., Saunders E., Leongamornlert D., Tymrakiewicz M., Mahmud N., Dadaev T., Govindasami K., Guy M. (2013). Germline BRCA mutations are associated with higher risk of nodal involvement, distant metastasis, and poor survival outcomes in prostate cancer. J. Clin. Oncol..

[6] Akbari M.R., Wallis C.J., Toi A., Trachtenberg J., Sun P., Narod S.A., Nam R.K. (2014). The impact of a BRCA2 mutation on mortality from screen-detected prostate cancer. Br. J. Cancer.

[7] Narod S.A., Neuhausen S., Vichodez G., Armel S., Lynch H.T., Ghadirian P., Cummings S., Olopade O., Stoppa-Lyonnet D., Couch F. (2008). Rapid progression of prostate cancer in men with a BRCA2 mutation. Br. J. Cancer.

[8] Lozano R., Salles D.C., Sandhu S., Aragón I.M., Thorne H., López-Campos F., Rubio-Briones J., Gutierrez-Pecharroman A.M., Maldonado L., di Domenico T. (2021). Association between BRCA2 alterations and intraductal and cribriform histologies in prostate cancer. Eur. J. Cancer.

[9] Pritchard C.C., Mateo J., Walsh M.F., De Sarkar N., Abida W., Beltran H., Garofalo A., Gulati R., Carreira S., Eeles R. (2016). Inherited DNA-Repair Gene Mutations in Men with Metastatic Prostate Cancer. N. Engl. J. Med..

[10] de Bono J., Mateo J., Fizazi K., Saad F., Shore N., Sandhu S., Chi K.N., Sartor O., Agarwal N., Olmos D. (2020). Olaparib for Metastatic Castration-Resistant Prostate Cancer. N. Engl. J. Med..

[11] Abida W., Patnaik A., Campbell D., Shapiro J., Bryce A.H., McDermott R., Sautois B., Vogelzang N.J., Bambury R.M., Voog E. (2020). Rucaparib in Men with Metastatic Castration-Resistant Prostate Cancer Harboring a BRCA1 or BRCA2 Gene Alteration. J. Clin. Oncol..

[12] de Bono J.S., Mehra N., Scagliotti G.V., Castro E., Dorff T., Stirling A., Stenzl A., Fleming M.T., Higano C.S., Saad F. (2021). Talazoparib monotherapy in metastatic castration-resistant prostate cancer with DNA repair alterations (TALAPRO-1): An open-label, phase 2 trial. Lancet Oncol..

[13] Clarke N., Wiechno P., Alekseev B., Sala N., Jones R., Kocak I., Chiuri V.E., Jassem J., Fléchon A., Redfern C. (2018). Olaparib combined with abiraterone in patients with metastatic castration-resistant prostate cancer: A randomised, double-blind, placebo-controlled, phase 2 trial. Lancet Oncol..

[14] Fizazi K., Piulats J.M., Reaume M.N., Ostler P., McDermott R., Gingerich J.R., Pintus E., Sridhar S.S., Bambury R.M., Emmenegger U. (2023). Rucaparib or Physician’s Choice in Metastatic Prostate Cancer. N. Engl. J. Med..

[15] Olivier M., Hollstein M., Hainaut P. (2010). TP53 mutations in human cancers: Origins, consequences, and clinical use. Cold Spring Harb. Perspect. Biol..

[16] The UniProt Consortium (2019). UniProt: A worldwide hub of protein knowledge. Nucleic Acids Res..

[17] Ng P.K., Li J., Jeong K.J., Shao S., Chen H., Tsang Y.H., Sengupta S., Wang Z., Bhavana V.H., Tran R. (2018). Systematic Functional Annotation of Somatic Mutations in Cancer. Cancer Cell.

[18] Forbes S., Clements J., Dawson E., Bamford S., Webb T., Dogan A., Flanagan A., Teague J., Wooster R., Futreal P.A. (2006). COSMIC 2005. Br. J. Cancer.

[19] Giri V.N., Hartman R., Pritzlaff M., Horton C., Keith S.W. (2022). Germline Variant Spectrum Among African American Men Undergoing Prostate Cancer Germline Testing: Need for Equity in Genetic Testing. JCO Precis. Oncol..

[20] Zurita A.J., Graf R.P., Villacampa G., Raskina K., Sokol E., Jin D., Antonarakis E.S., Li G., Huang R.S.P., Casanova-Salas I. (2022). Genomic Biomarkers and Genome-Wide Loss-of-Heterozygosity Scores in Metastatic Prostate Cancer Following Progression on Androgen-Targeting Therapies. JCO Precis. Oncol..

[21] Fonseca N.M., Van der Eecken K., Herberts C., Verbeke S., Ng S.W.S., Lumen N., Ritch E., Murtha A.J., Bernales C.Q., Schönlau E. (2022). Genomic Features of Lung-Recurrent Hormone-Sensitive Prostate Cancer. JCO Precis. Oncol..

[22] Gilson C., Ingleby F., Gilbert D.C., Parry M.A., Atako N.B., Ali A., Hoyle A., Clarke N.W., Gannon M., Wanstall C. (2020). Genomic Profiles of De Novo High- and Low-Volume Metastatic Prostate Cancer: Results From a 2-Stage Feasibility and Prevalence Study in the STAMPEDE Trial. JCO Precis. Oncol..

[23] Kimura H., Mizuno K., Shiota M., Narita S., Terada N., Fujimoto N., Ogura K., Hatano S., Iwasaki Y., Hakozaki N. (2022). Prognostic significance of pathogenic variants in BRCA1, BRCA2, ATM and PALB2 genes in men undergoing hormonal therapy for advanced prostate cancer. Br. J. Cancer.

[24] Castro E., Romero-Laorden N., Del Pozo A., Lozano R., Medina A., Puente J., Piulats J.M., Lorente D., Saez M.I., Morales-Barrera R. (2019). PROREPAIR-B: A Prospective Cohort Study of the Impact of Germline DNA Repair Mutations on the Outcomes of Patients with Metastatic Castration-Resistant Prostate Cancer. J. Clin. Oncol. Off. J. Am. Soc. Clin. Oncol..

[25] Price M.J., Vashistha V., Winski D., Kelley M.J., Bitting R.L., Montgomery B. (2022). Homologous Recombination Repair Gene Variants and Outcomes among Patients with Prostate Cancer Treated with Poly (ADP-ribose) Polymerase Inhibitors. JCO Precis. Oncol..

[26] Kyrochristos I.D., Glantzounis G.K., Goussia A., Eliades A., Achilleos A., Tsangaras K., Hadjidemetriou I., Elpidorou M., Ioannides M., Koumbaris G. (2022). Proof-of-Concept Pilot Study on Comprehensive Spatiotemporal Intra-Patient Heterogeneity for Colorectal Cancer with Liver Metastasis. Front. Oncol..

[27] Ikeda S., Elkin S.K., Tomson B.N., Carter J.L., Kurzrock R. (2019). Next-generation sequencing of prostate cancer: Genomic and pathway alterations, potential actionability patterns, and relative rate of use of clinical-grade testing. Cancer Biol. Ther..

[28] Ku S.Y., Gleave M.E., Beltran H. (2019). Towards precision oncology in advanced prostate cancer. Nat. Rev. Urol..

[29] Dawson N.A., Zibelman M., Lindsay T., Feldman R.A., Saul M., Gatalica Z., Korn W.M., Heath E.I. (2020). An Emerging Landscape for Canonical and Actionable Molecular Alterations in Primary and Metastatic Prostate Cancer. Mol. Cancer Ther..

[30] Papadopoulou K., Koliou G.A., Tsimiliotis D., Kotoula V., Foukas P., Goussia A., Tsiatas M., Visvikis A., Chatzopoulos K., Nifora M. (2022). Investigation of prognostic biomarkers in patients with urothelial carcinoma treated with platinum-based regimens. Urol. Oncol..

[31] Li H. (2013). Aligning sequence reads, clone sequences and assembly contigs with BWA-MEM. arXiv.

[32] Li H., Handsaker B., Wysoker A., Fennell T., Ruan J., Homer N., Marth G., Abecasis G., Durbin R. (2009). The Sequence Alignment/Map format and SAMtools. Bioinformatics.

[33] Lai Z., Markovets A., Ahdesmaki M., Chapman B., Hofmann O., McEwen R., Johnson J., Dougherty B., Barrett J.C., Dry J.R. (2016). VarDict: A novel and versatile variant caller for next-generation sequencing in cancer research. Nucleic Acids Res..

[34] McLaren W., Gil L., Hunt S.E., Riat H.S., Ritchie G.R., Thormann A., Flicek P., Cunningham F. (2016). The Ensembl Variant Effect Predictor. Genome Biol..

[35] Li M.M., Datto M., Duncavage E.J., Kulkarni S., Lindeman N.I., Roy S., Tsimberidou A.M., Vnencak-Jones C.L., Wolff D.J., Younes A. (2017). Standards and Guidelines for the Interpretation and Reporting of Sequence Variants in Cancer: A Joint Consensus Recommendation of the Association for Molecular Pathology, American Society of Clinical Oncology, and College of American Pathologists. J. Mol. Diagn..

[36] Tamborero D., Rubio-Perez C., Deu-Pons J., Schroeder M.P., Vivancos A., Rovira A., Tusquets I., Albanell J., Rodon J., Tabernero J. (2018). Cancer Genome Interpreter annotates the biological and clinical relevance of tumor alterations. Genome Med..

[37] Kopanos C., Tsiolkas V., Kouris A., Chapple C.E., Albarca Aguilera M., Meyer R., Massouras A. (2019). VarSome: The human genomic variant search engine. Bioinformatics.

[38] Seshan VE O.A. (2023). DNAcopy: DNA Copy Number Data Analysis.

[39] Layer R.M., Chiang C., Quinlan A.R., Hall I.M. (2014). LUMPY: A probabilistic framework for structural variant discovery. Genome Biol..

[40] Cameron D.L., Schröder J., Penington J.S., Do H., Molania R., Dobrovic A., Speed T.P., Papenfuss A.T. (2017). GRIDSS: Sensitive and specific genomic rearrangement detection using positional de Bruijn graph assembly. Genome Res..

[41] Wala J.A., Bandopadhayay P., Greenwald N.F., O’Rourke R., Sharpe T., Stewart C., Schumacher S., Li Y., Weischenfeldt J., Yao X. (2018). SvABA: Genome-wide detection of structural variants and indels by local assembly. Genome Res..

