# Supplementary material for: Investigation of Clinically Significant Molecular Aberrations in Patients with Prostate Cancer: Implications for Personalized Treatment, Prognosis and Genetic Testing
Source: Int J Mol Sci. 2023 Jul 23;24(14):11834. doi: 10.3390/ijms241411834 (PMC10380890; doi:10.3390/ijms241411834)

Supplemental Info

Supplemental Table S1. List of Genes covered by NIPD Genetics Prostate cancer assay.

Single Nucleotide Variants / Insertions and Deletions	Copy Number Alterations	Rearrangements
AKT1, APC, AR, ATM, BARD1, BRAF, BRCA1, BRCA2, CTNNB1, CHEK2, FOXA1, MLH1, MSH2, MSH6, NRAS, PALB2, PIK3CA, PIK3CB, PMS2, POLE, PTEN, RAD51C, RAD51D, SPOP, TP53	AR, ERBB2, FGFR1, FGFR2, FGFR3, MYC, MYCN, PIK3CA, PTEN, RB1, TP53	NTRK1, NTRK2, NTRK3, TMPRSS2
*Microsatellite Instability (MSI) testing included		

Supplemental Table S2. List of genomic coordinates covered by the Ampliseq panel (IAD207308_231).

Supplemental Table S3. List of all the clinically significant variants and MSI-H results identified in the cohort.

Supplemental Figure S1. Sanger sequencing analysis on 5 patients with suspected pathogenic/likely pathogenic germline variants in HRR genes.

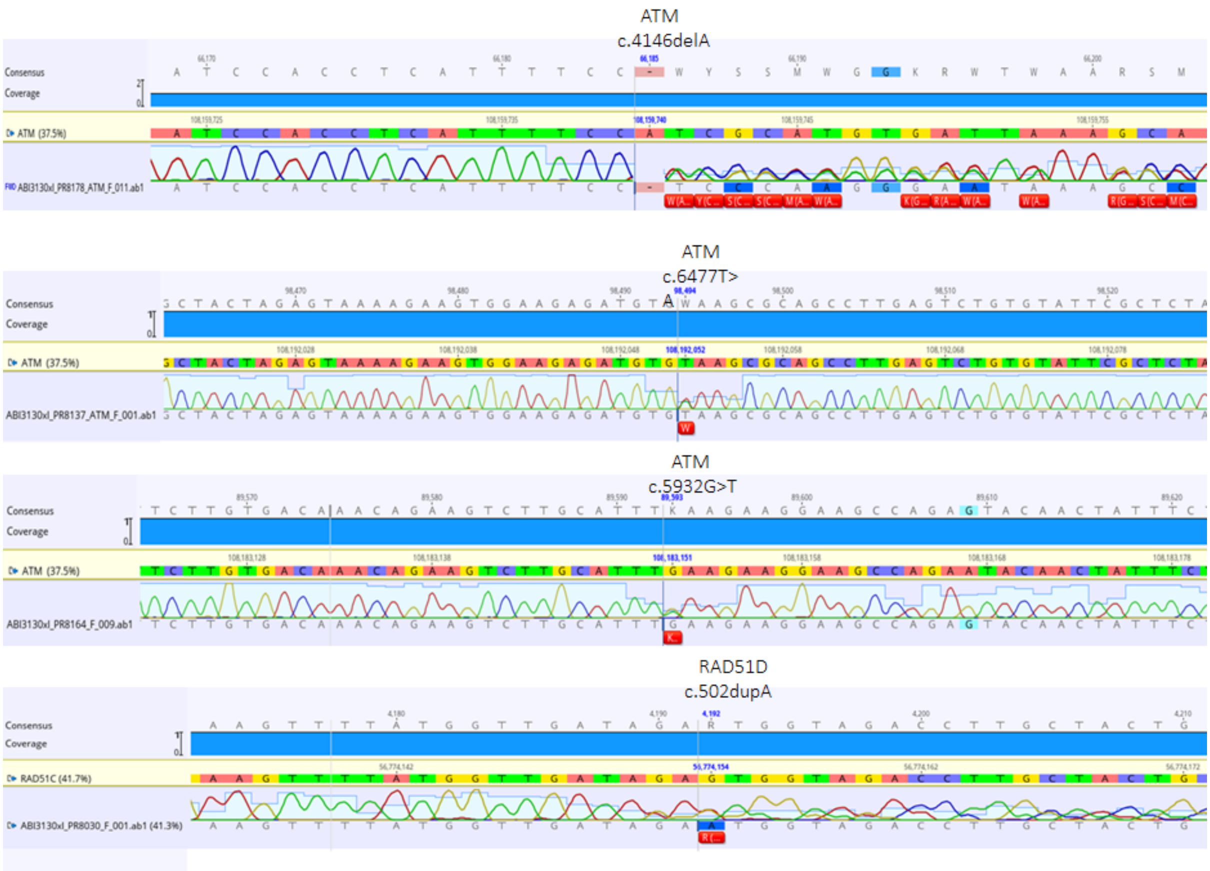

Supplement: Supplementary file 1 [file ijms-24-11834-s001.zip › Supplemental Info.pdf]
