# Supplementary material for: Investigation of Clinically Significant Molecular Aberrations in Patients with Prostate Cancer: Implications for Personalized Treatment, Prognosis and Genetic Testing
Source: Int J Mol Sci. 2023 Jul 23;24(14):11834. doi: 10.3390/ijms241411834 (PMC10380890; doi:10.3390/ijms241411834)
Supplement: Supplementary file 1 [file ijms-24-11834-s001.zip › Supplemental Table S2.pdf]

Amplicon ID	Chromosome	Amplicon start (hg19)	Amplicon end (hg19)
AMPL7170855335	chr1	27023549	27023683
AMPL7171869382	chr1	27023900	27024032
AMPL7170424868	chr1	27056347	27056470
AMPL7170424748	chr1	27057708	27057825
AMPL7171869517	chr1	27057974	27058095
AMPL7171812527	chr1	27087479	27087605
AMPL7170425568	chr1	27088716	27088837
AMPL7170424580	chr1	27089633	27089758
AMPL7171869369	chr1	27092769	27092894
AMPL7170623890	chr1	27097584	27097710
AMPL7170424591	chr1	27100076	27100205
AMPL7170425382	chr1	27100176	27100298
AMPL7171891882	chr1	27100895	27101013
AMPL7171902123	chr1	27101008	27101135
AMPL7171869116	chr1	27101159	27101283
AMPL7161412284	chr1	27101327	27101452
AMPL7170425442	chr1	27101637	27101765
AMPL7171869122	chr1	27105627	27105747
AMPL7171869000	chr1	27105758	27105854
AMPL7170086422	chr1	27105861	27105961
AMPL7171813124	chr1	27106482	27106597
AMPL7170424495	chr1	27106531	27106648
AMPL7170425472	chr1	27106751	27106860
AMPL7171869259	chr1	46714181	46714302
AMPL7171609162	chr1	46733135	46733261
AMPL7170596501	chr1	46736350	46736476
AMPL7170425219	chr1	46738104	46738226
AMPL7170582140	chr1	226561953	226562076
AMPL7171868796	chr1	226574071	226574182
AMPL7171869068	chr1	226576386	226576513
AMPL7170331871	chr10	89624206	89624333
AMPL7170691589	chr10	89624219	89624353
AMPL7171902011	chr10	89653752	89653852
AMPL7170333312	chr10	89653804	89653910
AMPL7171901954	chr10	89685207	89685313
AMPL7170334466	chr10	89685244	89685359
AMPL7171623896	chr10	89690783	89690891
AMPL7171902120	chr10	89692713	89692831
AMPL7171902129	chr10	89692816	89692944
AMPL7171901994	chr10	89692923	89693038
AMPL7171902060	chr10	89711810	89711936
AMPL7170336075	chr10	89711914	89712036
AMPL7170325896	chr10	89717571	89717693
AMPL7171224926	chr10	89717693	89717798
AMPL7170335369	chr10	89720578	89720695
AMPL7170325913	chr10	89720697	89720812
AMPL7170691519	chr10	89720801	89720916
AMPL7171891845	chr10	89720892	89721003

AMPL7170888696	chr10	89724846	89724922
AMPL7171902062	chr10	89724916	89725034
AMPL7170328563	chr10	89725017	89725129
AMPL7170623200	chr10	123239472	123239579
AMPL7170359373	chr10	123244865	123244961
AMPL7170359546	chr10	123246868	123246991
AMPL7170623683	chr10	123247444	123247559
AMPL7170088764	chr10	123255944	123256045
AMPL7170087909	chr10	123256041	123256144
AMPL7170359421	chr10	123257982	123258104
AMPL7170361230	chr10	123258041	123258160
AMPL7170361117	chr10	123263269	123263384
AMPL7171902021	chr10	123274779	123274901
AMPL7170624415	chr10	123276890	123277011
AMPL7170325903	chr10	123279413	123279539
AMPL7170325872	chr10	123279606	123279714
AMPL7170623674	chr10	123298138	123298265
AMPL7171137459	chr11	533377	533496
AMPL7171869013	chr11	533478	533616
AMPL7171902118	chr11	533799	533929
AMPL7170083455	chr11	534221	534307
AMPL7154679234	chr11	534264	534348
AMPL7171901935	chr11	22646295	22646419
AMPL7171891841	chr11	22646439	22646562
AMPL7170318298	chr11	69455970	69456111
AMPL7157266108	chr11	69456096	69456231
AMPL7157450846	chr11	69456221	69456334
AMPL7170319339	chr11	69457728	69457849
AMPL7171195852	chr11	69457870	69458008
AMPL7170319267	chr11	69457978	69458115
AMPL7170199804	chr11	69458605	69458706
AMPL7171902015	chr11	69458690	69458806
AMPL7171404332	chr11	69462778	69462887
AMPL7170202224	chr11	69462876	69462959
AMPL7171049851	chr11	69465777	69465911
AMPL7170318722	chr11	69465916	69466044
AMPL7171902040	chr11	94153277	94153401
AMPL7170326592	chr11	94178977	94179089
AMPL7170820990	chr11	94180358	94180483
AMPL7170326022	chr11	94189446	94189564
AMPL7171902127	chr11	94194090	94194182
AMPL7170111099	chr11	108098340	108098408
AMPL7170326319	chr11	108098396	108098525
AMPL7158273904	chr11	108098494	108098598
AMPL7152996649	chr11	108098577	108098691
AMPL7170326210	chr11	108099893	108099998
AMPL7170326014	chr11	108099958	108100080
AMPL7170326593	chr11	108106355	108106473
AMPL7169098460	chr11	108106470	108106573
AMPL7170308304	chr11	108114602	108114719

AMPL7170326261	chr11	108114706	108114829
AMPL7170804362	chr11	108114785	108114894
AMPL7170326562	chr11	108115505	108115625
AMPL7170326477	chr11	108115613	108115734
AMPL7170325978	chr11	108115728	108115846
AMPL7170308166	chr11	108117645	108117766
AMPL7170325641	chr11	108117714	108117836
AMPL7170325893	chr11	108117813	108117933
AMPL7170326697	chr11	108119587	108119714
AMPL7170326268	chr11	108119694	108119818
AMPL7170326604	chr11	108119765	108119885
AMPL7170325994	chr11	108121397	108121496
AMPL7170326698	chr11	108121483	108121603
AMPL7170804708	chr11	108121580	108121709
AMPL7170804527	chr11	108121687	108121807
AMPL7170326116	chr11	108122510	108122625
AMPL7170326437	chr11	108122618	108122739
AMPL7170082762	chr11	108122721	108122794
AMPL7170326362	chr11	108123519	108123625
AMPL7170326461	chr11	108123577	108123699
AMPL7162533182	chr11	108124514	108124635
AMPL7170308165	chr11	108124624	108124721
AMPL7170326504	chr11	108124707	108124829
AMPL7170308283	chr11	108126821	108126950
AMPL7170326077	chr11	108126956	108127056
AMPL7170308303	chr11	108127036	108127155
AMPL7170308198	chr11	108128202	108128319
AMPL7170326180	chr11	108128281	108128403
AMPL7170308203	chr11	108129700	108129805
AMPL7170308156	chr11	108129799	108129915
AMPL7157481509	chr11	108137891	108138012
AMPL7170325765	chr11	108137989	108138115
AMPL7170326429	chr11	108139082	108139192
AMPL7170804891	chr11	108139176	108139301
AMPL7170804667	chr11	108139236	108139359
AMPL7170804470	chr11	108141745	108141848
AMPL7170113475	chr11	108141828	108141920
AMPL7170326030	chr11	108141950	108142070
AMPL7170804932	chr11	108142056	108142176
AMPL7170326583	chr11	108143211	108143324
AMPL7170804382	chr11	108143311	108143421
AMPL7170113300	chr11	108143443	108143530
AMPL7170326541	chr11	108143494	108143616
AMPL7170326061	chr11	108150163	108150280
AMPL7170326700	chr11	108150255	108150369
AMPL7170326721	chr11	108151673	108151795
AMPL7170326465	chr11	108151795	108151912
AMPL7170199451	chr11	108153431	108153510
AMPL7170325997	chr11	108153496	108153605
AMPL7170308257	chr11	108153567	108153676

AMPL7170308307	chr11	108154887	108155005
AMPL7170804598	chr11	108154988	108155111
AMPL7170326498	chr11	108155093	108155205
AMPL7170325993	chr11	108158275	108158397
AMPL7170326086	chr11	108158373	108158494
AMPL7170804726	chr11	108159655	108159733
AMPL7170326226	chr11	108159719	108159838
AMPL7170334621	chr11	108160304	108160417
AMPL7170804778	chr11	108160385	108160485
AMPL7170326043	chr11	108160473	108160558
AMPL7163020447	chr11	108163275	108163397
AMPL7170804387	chr11	108163379	108163500
AMPL7170804810	chr11	108163448	108163565
AMPL7170804842	chr11	108163989	108164107
AMPL7170326195	chr11	108164075	108164178
AMPL7170804375	chr11	108164152	108164254
AMPL7170326488	chr11	108165630	108165752
AMPL7170326688	chr11	108165736	108165857
AMPL7170308270	chr11	108167997	108168122
AMPL7170325798	chr11	108170343	108170465
AMPL7170308346	chr11	108170454	108170578
AMPL7170326208	chr11	108170536	108170655
AMPL7170097240	chr11	108172361	108172456
AMPL7170325657	chr11	108172444	108172569
AMPL7170326421	chr11	108173541	108173642
AMPL7170097062	chr11	108173612	108173707
AMPL7170325902	chr11	108173693	108173815
AMPL7154394781	chr11	108175383	108175464
AMPL7170098208	chr11	108175434	108175525
AMPL7170326600	chr11	108175510	108175624
AMPL7170326564	chr11	108178582	108178703
AMPL7170326137	chr11	108178634	108178749
AMPL7170326296	chr11	108180781	108180899
AMPL7170326046	chr11	108180899	108180972
AMPL7170326385	chr11	108180960	108181077
AMPL7170308284	chr11	108183107	108183210
AMPL7170326423	chr11	108183159	108183280
AMPL7170326635	chr11	108186469	108186587
AMPL7170326454	chr11	108186555	108186671
AMPL7170326260	chr11	108186727	108186849
AMPL7170804474	chr11	108188070	108188184
AMPL7170326381	chr11	108188157	108188275
AMPL7170326598	chr11	108190626	108190721
AMPL7170326056	chr11	108190704	108190825
AMPL7155669633	chr11	108191948	108192075
AMPL7170804397	chr11	108192057	108192179
AMPL7170326018	chr11	108196028	108196151
AMPL7170326403	chr11	108196117	108196239
AMPL7170326236	chr11	108196228	108196337
AMPL7170804718	chr11	108196712	108196843

AMPL7170326016	chr11	108196842	108196961
AMPL7170326382	chr11	108198338	108198459
AMPL7170326005	chr11	108198380	108198498
AMPL7158863678	chr11	108199641	108199755
AMPL7170804712	chr11	108199749	108199860
AMPL7170804517	chr11	108199850	108199971
AMPL7170326653	chr11	108200928	108201044
AMPL7170326231	chr11	108201031	108201153
AMPL7170804582	chr11	108202089	108202214
AMPL7170308317	chr11	108202189	108202286
AMPL7170326596	chr11	108202280	108202399
AMPL7170804904	chr11	108202543	108202660
AMPL7170804820	chr11	108202648	108202769
AMPL7170326404	chr11	108203458	108203557
AMPL7170326517	chr11	108203545	108203659
AMPL7170325757	chr11	108204550	108204669
AMPL7170325655	chr11	108204652	108204772
AMPL7170308163	chr11	108205593	108205710
AMPL7170326446	chr11	108205707	108205793
AMPL7170326691	chr11	108205764	108205853
AMPL7170326069	chr11	108206521	108206644
AMPL7170326079	chr11	108206628	108206721
AMPL7170326073	chr11	108213917	108214036
AMPL7170326297	chr11	108214017	108214139
AMPL7170308237	chr11	108216397	108216514
AMPL7170326444	chr11	108216514	108216611
AMPL7170804559	chr11	108216580	108216703
AMPL7170325863	chr11	108217931	108218050
AMPL7170326368	chr11	108218023	108218144
AMPL7170308331	chr11	108224467	108224588
AMPL7170308246	chr11	108224526	108224638
AMPL7170325793	chr11	108225459	108225576
AMPL7170325768	chr11	108225565	108225689
AMPL7170326179	chr11	108235728	108235851
AMPL7170326040	chr11	108235834	108235951
AMPL7170326218	chr11	108235984	108236104
AMPL7170326578	chr11	108236069	108236188
AMPL7170326485	chr11	108236164	108236285
AMPL7170373534	chr11	125505333	125505453
AMPL7171894629	chr11	125513987	125514104
AMPL7170811027	chr11	125514050	125514153
AMPL7170374167	chr11	125525038	125525161
AMPL7171902103	chr11	125525185	125525306
AMPL7170318392	chr12	25368418	25368538
AMPL7170081672	chr12	25378540	25378612
AMPL7170081886	chr12	25380189	25380281
AMPL7170121181	chr12	25380260	25380364
AMPL7170308261	chr12	25398187	25398310
AMPL7170319135	chr12	25398271	25398388
AMPL7170545736	chr12	133208921	133209047

AMPL7171869541	chr12	133219911	133220040
AMPL7170373515	chr12	133241860	133241949
AMPL7170373302	chr12	133244926	133245050
AMPL7171869374	chr12	133249229	133249353
AMPL7170083264	chr12	133249750	133249829
AMPL7170623115	chr12	133249790	133249915
AMPL7170623389	chr12	133250049	133250187
AMPL7170624240	chr12	133250157	133250291
AMPL7170319362	chr12	133250274	133250377
AMPL7170319365	chr12	133251908	133252033
AMPL7170333331	chr12	133252000	133252137
AMPL7170545139	chr12	133252116	133252225
AMPL7171869316	chr12	133252239	133252374
AMPL7170623286	chr12	133252327	133252452
AMPL7170319363	chr12	133252601	133252725
AMPL7170319358	chr12	133252678	133252800
AMPL7170332667	chr12	133252779	133252904
AMPL7170623724	chr12	133252941	133253068
AMPL7170319368	chr12	133253038	133253160
AMPL7170088191	chr12	133253156	133253253
AMPL7170329140	chr12	133253221	133253337
AMPL7170318813	chr13	32890530	32890652
AMPL7154368026	chr13	32890603	32890727
AMPL7170318615	chr13	32893198	32893316
AMPL7170318104	chr13	32893273	32893381
AMPL7170318688	chr13	32893366	32893483
AMPL7170318189	chr13	32899201	32899308
AMPL7170318911	chr13	32899290	32899414
AMPL7170319263	chr13	32900249	32900373
AMPL7170319118	chr13	32900301	32900412
AMPL7170318040	chr13	32900410	32900529
AMPL7170334658	chr13	32900559	32900678
AMPL7170330042	chr13	32900659	32900780
AMPL7170318334	chr13	32903522	32903637
AMPL7170331981	chr13	32903614	32903733
AMPL7170318121	chr13	32905020	32905140
AMPL7170329305	chr13	32905122	32905244
AMPL7170318458	chr13	32906336	32906453
AMPL7170318258	chr13	32906426	32906515
AMPL7170319013	chr13	32906512	32906610
AMPL7170319181	chr13	32906601	32906684
AMPL7170318243	chr13	32906654	32906771
AMPL7170319117	chr13	32906762	32906868
AMPL7170318758	chr13	32906858	32906981
AMPL7157781722	chr13	32906960	32907067
AMPL7170319164	chr13	32907051	32907172
AMPL7170319309	chr13	32907168	32907284
AMPL7170318200	chr13	32907230	32907336
AMPL7170318135	chr13	32907335	32907461
AMPL7170319351	chr13	32907453	32907561

AMPL7170318226	chr13	32910386	32910454
AMPL7170318378	chr13	32910423	32910512
AMPL7170318402	chr13	32910497	32910605
AMPL7170318242	chr13	32910570	32910671
AMPL7170319146	chr13	32910648	32910772
AMPL7170319001	chr13	32910763	32910852
AMPL7170318347	chr13	32910819	32910934
AMPL7170318446	chr13	32910900	32911017
AMPL7170319338	chr13	32910997	32911118
AMPL7170318626	chr13	32911077	32911189
AMPL7170319208	chr13	32911186	32911296
AMPL7170318841	chr13	32911251	32911370
AMPL7170318912	chr13	32911352	32911472
AMPL7170318814	chr13	32911421	32911519
AMPL7170318262	chr13	32911519	32911620
AMPL7170318050	chr13	32911571	32911675
AMPL7170318868	chr13	32911670	32911783
AMPL7170318229	chr13	32911781	32911888
AMPL7170318274	chr13	32911831	32911946
AMPL7170318917	chr13	32911934	32912017
AMPL7170311679	chr13	32911989	32912065
AMPL7170318113	chr13	32912065	32912173
AMPL7170318517	chr13	32912109	32912234
AMPL7170318284	chr13	32912218	32912287
AMPL7170318848	chr13	32912278	32912373
AMPL7170085939	chr13	32912403	32912467
AMPL7170318068	chr13	32912424	32912533
AMPL7170085454	chr13	32912517	32912593
AMPL7170319201	chr13	32912581	32912659
AMPL7170085046	chr13	32912638	32912712
AMPL7170318501	chr13	32912698	32912802
AMPL7170318600	chr13	32912802	32912916
AMPL7164384001	chr13	32912864	32912986
AMPL7170318946	chr13	32912980	32913058
AMPL7170318655	chr13	32913034	32913158
AMPL7170085048	chr13	32913125	32913201
AMPL7170318445	chr13	32913197	32913309
AMPL7170085086	chr13	32913247	32913333
AMPL7170318588	chr13	32913345	32913430
AMPL7170318027	chr13	32913392	32913490
AMPL7170318586	chr13	32913469	32913567
AMPL7170319329	chr13	32913545	32913650
AMPL7170318157	chr13	32913618	32913716
AMPL7170318545	chr13	32913703	32913790
AMPL7170318472	chr13	32913759	32913865
AMPL7170085749	chr13	32913836	32913923
AMPL7170319168	chr13	32913912	32913992
AMPL7167079445	chr13	32913966	32914036
AMPL7170319175	chr13	32914036	32914163
AMPL7170319033	chr13	32914153	32914237

AMPL7170319315	chr13	32914204	32914291
AMPL7170319060	chr13	32914287	32914399
AMPL7170319023	chr13	32914355	32914476
AMPL7170319297	chr13	32914444	32914546
AMPL7170319010	chr13	32914528	32914645
AMPL7155551814	chr13	32914587	32914710
AMPL7170318695	chr13	32914690	32914814
AMPL7170318770	chr13	32914791	32914895
AMPL7170319230	chr13	32914860	32914982
AMPL7170318975	chr13	32914976	32915047
AMPL7170319180	chr13	32915043	32915161
AMPL7170318802	chr13	32915108	32915228
AMPL7170425658	chr13	32915224	32915346
AMPL7170318510	chr13	32918652	32918769
AMPL7170318882	chr13	32918719	32918813
AMPL7170318522	chr13	32920879	32920996
AMPL7170086693	chr13	32920981	32921054
AMPL7170319300	chr13	32929017	32929108
AMPL7170318723	chr13	32929068	32929178
AMPL7170318854	chr13	32929157	32929274
AMPL7170318967	chr13	32929260	32929379
AMPL7170318152	chr13	32929319	32929441
AMPL7170319212	chr13	32930589	32930687
AMPL7170085706	chr13	32930673	32930765
AMPL7170318938	chr13	32931804	32931921
AMPL7170318691	chr13	32931908	32932032
AMPL7170319073	chr13	32931991	32932110
AMPL7170318565	chr13	32936626	32936748
AMPL7170318701	chr13	32936748	32936866
AMPL7170318352	chr13	32937283	32937404
AMPL7170318834	chr13	32937384	32937503
AMPL7170318653	chr13	32937479	32937591
AMPL7170318781	chr13	32937559	32937680
AMPL7170318618	chr13	32944471	32944584
AMPL7170318622	chr13	32944558	32944642
AMPL7170318772	chr13	32944624	32944743
AMPL7170318464	chr13	32945051	32945162
AMPL7170318476	chr13	32945147	32945252
AMPL7170318206	chr13	32950730	32950853
AMPL7170318580	chr13	32950837	32950951
AMPL7170318651	chr13	32953387	32953505
AMPL7170318408	chr13	32953465	32953583
AMPL7170318049	chr13	32953551	32953646
AMPL7170318828	chr13	32953634	32953755
AMPL7170331482	chr13	32953784	32953899
AMPL7157782884	chr13	32953880	32954002
AMPL7170319334	chr13	32953972	32954084
AMPL7170318523	chr13	32954111	32954230
AMPL7170319302	chr13	32954218	32954332
AMPL7170318101	chr13	32968781	32968880

AMPL7170318713	chr13	32968875	32968996
AMPL7170319078	chr13	32968979	32969085
AMPL7170319050	chr13	32971018	32971141
AMPL7170319346	chr13	32971102	32971223
AMPL7170319104	chr13	32972286	32972401
AMPL7170318890	chr13	32972366	32972489
AMPL7170318703	chr13	32972476	32972598
AMPL7170318465	chr13	32972586	32972707
AMPL7170318955	chr13	32972690	32972813
AMPL7170318187	chr13	32972807	32972922
AMPL7170422410	chr13	48878051	48878180
AMPL7170080842	chr13	48878144	48878238
AMPL7170421748	chr13	48881428	48881528
AMPL7171901972	chr13	48916693	48916799
AMPL7171902097	chr13	48916817	48916901
AMPL7171891864	chr13	48919276	48919377
AMPL7170081077	chr13	48921923	48921998
AMPL7170622197	chr13	48921968	48922084
AMPL7171902017	chr13	48923035	48923131
AMPL7169638873	chr13	48923120	48923212
AMPL7171902065	chr13	48934058	48934175
AMPL7170559101	chr13	48934126	48934240
AMPL7170420909	chr13	48936957	48937062
AMPL7170624792	chr13	48937021	48937128
AMPL7171891922	chr13	48938970	48939096
AMPL7171901924	chr13	48939031	48939150
AMPL7170875789	chr13	48941590	48941710
AMPL7170647023	chr13	48941654	48941760
AMPL7170558825	chr13	48947553	48947661
AMPL7171902088	chr13	48951030	48951152
AMPL7171902130	chr13	48951143	48951223
AMPL7170104478	chr13	48953705	48953770
AMPL7171891908	chr13	48954199	48954309
AMPL7170420947	chr13	48954319	48954410
AMPL7170424325	chr13	48955359	48955474
AMPL7171902114	chr13	48955451	48955569
AMPL7170325862	chr13	49027095	49027200
AMPL7170202534	chr13	49027192	49027279
AMPL7170420618	chr13	49030317	49030438
AMPL7171574538	chr13	49030411	49030530
AMPL7170325678	chr13	49033831	49033952
AMPL7170325842	chr13	49037844	49037961
AMPL7162580182	chr13	49037944	49038051
AMPL7170104960	chr13	49039143	49039240
AMPL7170423410	chr13	49039261	49039387
AMPL7170422263	chr13	49039366	49039491
AMPL7170423569	chr13	49047404	49047519
AMPL7170421355	chr13	49050795	49050912
AMPL7169707799	chr13	103506617	103506703
AMPL7170595632	chr13	103508386	103508494

AMPL7170596598	chr13	103515030	103515145
AMPL7171868824	chr13	103519070	103519183
AMPL7170812178	chr13	103524521	103524619
AMPL7170595722	chr13	103524602	103524720
AMPL7171869375	chr13	103527591	103527714
AMPL7170373844	chr14	68301813	68301933
AMPL7170287075	chr14	68331738	68331837
AMPL7171894627	chr14	68331824	68331916
AMPL7171901971	chr14	68352599	68352692
AMPL7170816298	chr14	68353800	68353905
AMPL7170821163	chr14	68353885	68354007
AMPL7170374381	chr14	68758564	68758686
AMPL7170808268	chr14	68878092	68878217
AMPL7170374393	chr14	69061303	69061428
AMPL7171901977	chr14	105236655	105236755
AMPL7170832245	chr14	105237139	105237242
AMPL7171902045	chr14	105239114	105239227
AMPL7171033520	chr14	105239329	105239463
AMPL7171902113	chr14	105239567	105239702
AMPL7171049737	chr14	105239853	105239983
AMPL7170319020	chr14	105241323	105241450
AMPL7170876413	chr14	105242033	105242165
AMPL7170319239	chr14	105242948	105243085
AMPL7170318313	chr14	105243063	105243198
AMPL7170176225	chr14	105246406	105246506
AMPL7170479882	chr14	105246470	105246586
AMPL7170326400	chr15	41020920	41021042
AMPL7162579763	chr15	89816598	89816714
AMPL7171033572	chr15	89828339	89828457
AMPL7171033083	chr15	89834864	89834987
AMPL7171652691	chr15	89836119	89836239
AMPL7171891933	chr15	89837168	89837292
AMPL7171651702	chr15	89848380	89848492
AMPL7171033503	chr15	89857893	89858013
AMPL7171033238	chr15	89859605	89859729
AMPL7163715862	chr15	91303401	91303491
AMPL7171869214	chr15	91304067	91304179
AMPL7170807867	chr15	91304183	91304302
AMPL7170365439	chr15	91312645	91312768
AMPL7170806324	chr15	91333843	91333967
AMPL7170365278	chr15	91337432	91337551
AMPL7170364908	chr16	23614760	23614856
AMPL7170332101	chr16	23614844	23614959
AMPL7170332699	chr16	23614928	23615053
AMPL7170425508	chr16	23619164	23619275
AMPL7170336370	chr16	23619262	23619383
AMPL7170336146	chr16	23625257	23625378
AMPL7170328941	chr16	23625363	23625489
AMPL7170333835	chr16	23632631	23632750
AMPL7170333670	chr16	23632697	23632817

AMPL7170330720	chr16	23634245	23634367
AMPL7170334095	chr16	23634339	23634463
AMPL7170425176	chr16	23635315	23635432
AMPL7170333014	chr16	23637525	23637653
AMPL7170330469	chr16	23637629	23637754
AMPL7170333085	chr16	23640506	23640627
AMPL7170334146	chr16	23640907	23641020
AMPL7170331461	chr16	23641015	23641135
AMPL7170333461	chr16	23641091	23641215
AMPL7170334206	chr16	23641201	23641330
AMPL7170335433	chr16	23641286	23641411
AMPL7170336194	chr16	23641398	23641521
AMPL7170333454	chr16	23641497	23641621
AMPL7170335445	chr16	23641598	23641713
AMPL7170328920	chr16	23641701	23641822
AMPL7170332238	chr16	23646137	23646261
AMPL7170330715	chr16	23646241	23646361
AMPL7170334114	chr16	23646349	23646469
AMPL7170328583	chr16	23646443	23646555
AMPL7170328665	chr16	23646514	23646625
AMPL7170332435	chr16	23646621	23646733
AMPL7170334075	chr16	23646704	23646785
AMPL7170334365	chr16	23646778	23646900
AMPL7170328766	chr16	23646856	23646954
AMPL7170335267	chr16	23646949	23647049
AMPL7170334113	chr16	23647023	23647136
AMPL7170330211	chr16	23647099	23647226
AMPL7170328950	chr16	23647205	23647329
AMPL7170146976	chr16	23647297	23647390
AMPL7162679764	chr16	23647380	23647504
AMPL7170334060	chr16	23647503	23647603
AMPL7170145563	chr16	23647555	23647635
AMPL7170330639	chr16	23647664	23647780
AMPL7162997616	chr16	23649150	23649270
AMPL7170330832	chr16	23649268	23649376
AMPL7170146670	chr16	23649380	23649468
AMPL7170146089	chr16	23652389	23652489
AMPL7169939244	chr16	89806403	89806505
AMPL7170373571	chr16	89807216	89807335
AMPL7170374316	chr16	89812906	89813037
AMPL7170374064	chr16	89813168	89813290
AMPL7170374239	chr16	89816083	89816212
AMPL7171725711	chr16	89818529	89818655
AMPL7171891855	chr16	89828353	89828455
AMPL7170373905	chr16	89831272	89831384
AMPL7171891867	chr16	89831319	89831442
AMPL7170373605	chr16	89833510	89833629
AMPL7170287349	chr16	89838137	89838234
AMPL7170373602	chr16	89849218	89849340
AMPL7170373993	chr16	89851302	89851435

AMPL7170374101	chr16	89869652	89869776
AMPL7170287320	chr16	89874678	89874758
AMPL7170374343	chr16	89877073	89877189
AMPL7171653202	chr16	89877352	89877474
AMPL7170309695	chr17	7572852	7572977
AMPL7170309709	chr17	7572930	7573030
AMPL7171365824	chr17	7573875	7574003
AMPL7170309708	chr17	7573931	7574045
AMPL7170309701	chr17	7576494	7576592
AMPL7170309705	chr17	7576564	7576687
AMPL7153774031	chr17	7576828	7576949
AMPL7171365826	chr17	7577006	7577137
AMPL7171365820	chr17	7577080	7577197
AMPL7170836823	chr17	7577485	7577622
AMPL7170309710	chr17	7578142	7578261
AMPL7170623892	chr17	7578240	7578363
AMPL7171365823	chr17	7578309	7578411
AMPL7171365825	chr17	7578397	7578518
AMPL7171365822	chr17	7578445	7578575
AMPL7171365821	chr17	7579276	7579395
AMPL7170309693	chr17	7579372	7579506
AMPL7170327659	chr17	7579451	7579582
AMPL7170327642	chr17	7579576	7579701
AMPL7170327922	chr17	7579618	7579732
AMPL7170309720	chr17	7579785	7579922
AMPL7170365629	chr17	33428153	33428279
AMPL7171894632	chr17	33428233	33428366
AMPL7171894626	chr17	33430476	33430606
AMPL7170365359	chr17	33434031	33434158
AMPL7171869401	chr17	33434432	33434557
AMPL7171901952	chr17	33443832	33443957
AMPL7170332225	chr17	33446557	33446671
AMPL7170425492	chr17	37618285	37618422
AMPL7170425309	chr17	37618387	37618514
AMPL7170425518	chr17	37618484	37618616
AMPL7170425426	chr17	37618603	37618734
AMPL7170425435	chr17	37618697	37618787
AMPL7170425081	chr17	37618773	37618902
AMPL7170425724	chr17	37618858	37618969
AMPL7170425265	chr17	37618963	37619086
AMPL7170425455	chr17	37619078	37619203
AMPL7170424891	chr17	37619130	37619256
AMPL7170424468	chr17	37619243	37619368
AMPL7170425216	chr17	37619315	37619432
AMPL7170424638	chr17	37627117	37627222
AMPL7170425118	chr17	37627158	37627279
AMPL7170425597	chr17	37627263	37627388
AMPL7170424734	chr17	37627363	37627471
AMPL7170424427	chr17	37627437	37627558
AMPL7170424378	chr17	37627541	37627665

AMPL7170424590	chr17	37627617	37627743
AMPL7170424508	chr17	37627714	37627840
AMPL7170424657	chr17	37627821	37627948
AMPL7170425287	chr17	37627928	37628045
AMPL7170425444	chr17	37646742	37646867
AMPL7170425134	chr17	37646856	37646976
AMPL7170424717	chr17	37646976	37647081
AMPL7170424893	chr17	37648981	37649101
AMPL7158542910	chr17	37649032	37649151
AMPL7170425547	chr17	37650729	37650849
AMPL7170752249	chr17	37650830	37650952
AMPL7170424552	chr17	37657456	37657580
AMPL7166736788	chr17	37657514	37657635
AMPL7170425516	chr17	37657624	37657731
AMPL7170425689	chr17	37665911	37666029
AMPL7170424646	chr17	37667758	37667878
AMPL7170425170	chr17	37667855	37667980
AMPL7170425708	chr17	37671976	37672094
AMPL7170425254	chr17	37673644	37673773
AMPL7170425459	chr17	37673749	37673876
AMPL7170424921	chr17	37676181	37676308
AMPL7170424944	chr17	37676233	37676359
AMPL7170424448	chr17	37680916	37681038
AMPL7170424658	chr17	37681033	37681160
AMPL7170840454	chr17	37682047	37682159
AMPL7170838904	chr17	37682138	37682268
AMPL7170839929	chr17	37682248	37682380
AMPL7170838671	chr17	37682353	37682482
AMPL7170424758	chr17	37682451	37682575
AMPL7170839306	chr17	37686851	37686976
AMPL7170840408	chr17	37686955	37687066
AMPL7170840825	chr17	37687050	37687183
AMPL7170839902	chr17	37687164	37687294
AMPL7170645696	chr17	37687278	37687413
AMPL7170646084	chr17	37687390	37687519
AMPL7170839868	chr17	37687504	37687611
AMPL7170624475	chr17	37868166	37868259
AMPL7170623688	chr17	37879547	37879674
AMPL7170318489	chr17	37879709	37879844
AMPL7157484523	chr17	37879812	37879907
AMPL7170692061	chr17	37879884	37879972
AMPL7170319222	chr17	37880117	37880237
AMPL7170319328	chr17	37880224	37880354
AMPL7170559049	chr17	37880961	37881092
AMPL7170318295	chr17	37881080	37881207
AMPL7171902125	chr17	37881290	37881420
AMPL7170318033	chr17	37881389	37881503
AMPL7170318267	chr17	37881521	37881647
AMPL7170203617	chr17	37881642	37881730
AMPL7170304558	chr17	37881915	37881993

AMPL7170318572	chr17	37881965	37882093
AMPL7170318201	chr17	37882087	37882212
AMPL7170318795	chr17	37882738	37882852
AMPL7170448811	chr17	37882863	37882973
AMPL7170318453	chr17	41197683	41197817
AMPL7170860656	chr17	41197773	41197877
AMPL7170087639	chr17	41199642	41199741
AMPL7170318349	chr17	41201118	41201241
AMPL7170318717	chr17	41203047	41203164
AMPL7170318872	chr17	41209055	41209177
AMPL7170860650	chr17	41215289	41215409
AMPL7170860728	chr17	41215866	41215983
AMPL7170318148	chr17	41219609	41219730
AMPL7170860576	chr17	41222930	41223048
AMPL7170860620	chr17	41223027	41223158
AMPL7170860555	chr17	41223147	41223264
AMPL7170319039	chr17	41226340	41226464
AMPL7170318418	chr17	41226437	41226565
AMPL7170860603	chr17	41228489	41228583
AMPL7170860554	chr17	41228553	41228665
AMPL7170318684	chr17	41231313	41231431
AMPL7170318289	chr17	41234413	41234526
AMPL7170319082	chr17	41234495	41234607
AMPL7170318815	chr17	41242918	41243038
AMPL7170318839	chr17	41242971	41243090
AMPL7170318440	chr17	41243392	41243515
AMPL7170318299	chr17	41243504	41243627
AMPL7170318726	chr17	41243611	41243730
AMPL7170318900	chr17	41243727	41243846
AMPL7170319220	chr17	41243832	41243954
AMPL7170318154	chr17	41243941	41244065
AMPL7170318301	chr17	41244054	41244168
AMPL7170319110	chr17	41244139	41244263
AMPL7170318507	chr17	41244257	41244375
AMPL7170318944	chr17	41244367	41244486
AMPL7170318168	chr17	41244486	41244610
AMPL7157781194	chr17	41244609	41244735
AMPL7158722897	chr17	41244730	41244847
AMPL7170318373	chr17	41244832	41244950
AMPL7170319144	chr17	41244935	41245051
AMPL7170318098	chr17	41245044	41245170
AMPL7170318420	chr17	41245169	41245292
AMPL7158524919	chr17	41245286	41245409
AMPL7170318776	chr17	41245404	41245525
AMPL7170319253	chr17	41245504	41245624
AMPL7170319244	chr17	41245590	41245712
AMPL7170328456	chr17	41245683	41245802
AMPL7170333152	chr17	41245784	41245907
AMPL7170330034	chr17	41245895	41246016
AMPL7170331826	chr17	41245985	41246111

AMPL7170864073	chr17	41246086	41246187
AMPL7170623742	chr17	41246160	41246284
AMPL7170318395	chr17	41246268	41246390
AMPL7170319169	chr17	41246368	41246490
AMPL7170318268	chr17	41246472	41246587
AMPL7170319142	chr17	41246565	41246694
AMPL7170319011	chr17	41246666	41246791
AMPL7170318730	chr17	41246778	41246895
AMPL7170318138	chr17	41247846	41247967
AMPL7170319322	chr17	41249249	41249367
AMPL7170860619	chr17	41251754	41251866
AMPL7170860686	chr17	41251822	41251944
AMPL7170860594	chr17	41256049	41256182
AMPL7170318752	chr17	41256161	41256283
AMPL7170318060	chr17	41256868	41256992
AMPL7170082182	chr17	41258441	41258526
AMPL7170860721	chr17	41258512	41258631
AMPL7170860626	chr17	41267686	41267785
AMPL7170318590	chr17	41267763	41267885
AMPL7170318893	chr17	41276006	41276121
AMPL7172214180	chr17	46805577	46805702
AMPL7172214181	chr17	46805696	46805809
AMPL7171894647	chr17	56770033	56770148
AMPL7170334848	chr17	56772452	56772561
AMPL7170335064	chr17	56774091	56774211
AMPL7170146882	chr17	56787231	56787320
AMPL7170328666	chr17	56798097	56798208
AMPL7171869027	chr17	59760747	59760852
AMPL7170329095	chr17	59760824	59760942
AMPL7170333536	chr17	59760921	59761023
AMPL7171902090	chr17	59761279	59761399
AMPL7171901981	chr17	59761402	59761522
AMPL7171653482	chr17	59763231	59763350
AMPL7170145978	chr17	59763463	59763541
AMPL7171901982	chr17	59793320	59793394
AMPL7171891940	chr17	59820330	59820451
AMPL7170335745	chr17	59853688	59853812
AMPL7170331174	chr17	59858170	59858278
AMPL7170424989	chr17	59858302	59858405
AMPL7170328364	chr17	59861643	59861762
AMPL7171901953	chr17	59876449	59876572
AMPL7171902071	chr17	59878633	59878727
AMPL7171901992	chr17	59878750	59878849
AMPL7171901957	chr17	59885903	59886024
AMPL7169918808	chr17	59886034	59886117
AMPL7170365675	chr17	59924453	59924558
AMPL7170331890	chr17	59926450	59926574
AMPL7170328635	chr17	59937099	59937223
AMPL7171902098	chr17	59938844	59938967
AMPL7170595856	chr19	45855430	45855536

AMPL7170595651	chr19	45855710	45855838
AMPL7170596160	chr19	45855792	45855913
AMPL7171366107	chr19	45855880	45855987
AMPL7169255233	chr19	45856012	45856104
AMPL7170819376	chr19	45856350	45856438
AMPL7170818707	chr19	45860949	45861078
AMPL7171891935	chr19	45868395	45868519
AMPL7159089280	chr19	45873426	45873556
AMPL7170595989	chr19	45873749	45873840
AMPL7171451107	chr19	45916741	45916864
AMPL7170808094	chr19	45917228	45917312
AMPL7171869324	chr19	45918188	45918289
AMPL7171868987	chr19	45924355	45924450
AMPL7171869029	chr2	25457167	25457264
AMPL7171673257	chr2	25464446	25464529
AMPL7171869317	chr2	25469049	25469166
AMPL7171869254	chr2	25469169	25469299
AMPL7171869505	chr2	25470425	25470525
AMPL7170621388	chr2	25470962	25471093
AMPL7171380690	chr2	47630401	47630516
AMPL7170088060	chr2	47630506	47630599
AMPL7171894616	chr2	47635508	47635618
AMPL7157780050	chr2	47635559	47635660
AMPL7170332414	chr2	47637250	47637377
AMPL7171901938	chr2	47637334	47637445
AMPL7171208453	chr2	47637456	47637557
AMPL7170328215	chr2	47639523	47639627
AMPL7171902109	chr2	47639582	47639682
AMPL7171869120	chr2	47639683	47639801
AMPL7171894648	chr2	47641382	47641482
AMPL7170331380	chr2	47641426	47641533
AMPL7170334766	chr2	47643407	47643529
AMPL7171902047	chr2	47656797	47656911
AMPL7171902073	chr2	47656912	47657019
AMPL7170335597	chr2	47656997	47657120
AMPL7171902022	chr2	47672613	47672733
AMPL7170480679	chr2	47672738	47672842
AMPL7170329555	chr2	47690169	47690292
AMPL7170181398	chr2	47693823	47693930
AMPL7170328809	chr2	47698128	47698227
AMPL7170333913	chr2	47702080	47702197
AMPL7171902069	chr2	47702217	47702290
AMPL7171894644	chr2	47702266	47702370
AMPL7160675561	chr2	47703427	47703551
AMPL7170332477	chr2	47703529	47703649
AMPL7171894670	chr2	47703671	47703757
AMPL7170365067	chr2	47705490	47705616
AMPL7170331415	chr2	47707837	47707962
AMPL7171901959	chr2	47707890	47708005
AMPL7171869002	chr2	47709859	47709961

AMPL7170334500	chr2	48018028	48018157
AMPL7170334916	chr2	48018194	48018320
AMPL7170331422	chr2	48023022	48023143
AMPL7171902070	chr2	48025743	48025820
AMPL7171901933	chr2	48025822	48025941
AMPL7171902028	chr2	48025930	48026049
AMPL7171902009	chr2	48026113	48026226
AMPL7171894657	chr2	48026249	48026327
AMPL7171901931	chr2	48026351	48026457
AMPL7170331289	chr2	48026436	48026559
AMPL7171902037	chr2	48026579	48026704
AMPL7162606051	chr2	48026835	48026956
AMPL7171902007	chr2	48026988	48027095
AMPL7170088862	chr2	48027073	48027172
AMPL7170328274	chr2	48027302	48027429
AMPL7171869432	chr2	48027715	48027838
AMPL7171728442	chr2	48027765	48027890
AMPL7171894638	chr2	48027892	48027978
AMPL7157782595	chr2	48028001	48028126
AMPL7170329189	chr2	48030523	48030650
AMPL7171401927	chr2	48030637	48030760
AMPL7170818007	chr2	48032035	48032147
AMPL7170365811	chr2	48033357	48033478
AMPL7170328313	chr2	48033445	48033551
AMPL7170365779	chr2	48033644	48033764
AMPL7171902041	chr2	48033739	48033840
AMPL7171869052	chr2	58386921	58387040
AMPL7171055439	chr2	58388626	58388723
AMPL7171055452	chr2	58390576	58390687
AMPL7171055449	chr2	58431294	58431414
AMPL7171600198	chr2	58459198	58459297
AMPL7171868844	chr2	190682720	190682843
AMPL7170595999	chr2	190717341	190717462
AMPL7170596145	chr2	190718612	190718734
AMPL7171869526	chr2	190718943	190719050
AMPL7159089783	chr2	190719477	190719596
AMPL7171869558	chr2	190728663	190728787
AMPL7171902027	chr2	215595052	215595166
AMPL7171869402	chr2	215595150	215595264
AMPL7171902095	chr2	215645463	215645573
AMPL7171891944	chr2	215645703	215645822
AMPL7171902086	chr2	215645864	215645988
AMPL7171891877	chr2	215646012	215646138
AMPL7170335942	chr2	215657026	215657145
AMPL7170326323	chr22	29083967	29084097
AMPL7170326033	chr22	29089946	29090052
AMPL7170326001	chr22	29090040	29090159
AMPL7170326311	chr22	29091081	29091211
AMPL7170326127	chr22	29091142	29091270
AMPL7170326353	chr22	29091676	29091805

AMPL7170326105	chr22	29091789	29091923
AMPL7170326369	chr22	29092876	29092996
AMPL7170326251	chr22	29095746	29095866
AMPL7170326000	chr22	29095856	29095972
AMPL7153201261	chr22	29099461	29099584
AMPL7170091413	chr22	29105951	29106041
AMPL7163020393	chr22	29106034	29106146
AMPL7170111315	chr22	29107879	29107952
AMPL7170326730	chr22	29107894	29108011
AMPL7170425609	chr22	29107997	29108114
AMPL7170111822	chr22	29115332	29115421
AMPL7170326438	chr22	29115408	29115516
AMPL7170326052	chr22	29120922	29121043
AMPL7170326168	chr22	29121003	29121123
AMPL7170326151	chr22	29121207	29121329
AMPL7170326245	chr22	29121309	29121431
AMPL7170118411	chr22	29126411	29126515
AMPL7170336028	chr22	29130380	29130494
AMPL7170325968	chr22	29130481	29130594
AMPL7170335153	chr22	29130584	29130712
AMPL7170336255	chr22	29130694	29130784
AMPL7171224802	chr22	30057140	30057246
AMPL7170305164	chr22	30067801	30067926
AMPL7170374203	chr3	10083278	10083397
AMPL7170374315	chr3	10101920	10102033
AMPL7170374270	chr3	10105394	10105522
AMPL7170373915	chr3	10106024	10106150
AMPL7171651934	chr3	10108897	10109003
AMPL7171891840	chr3	10116212	10116336
AMPL7170374060	chr3	10128791	10128903
AMPL7170334512	chr3	37035048	37035179
AMPL7171901985	chr3	37038012	37038135
AMPL7170546080	chr3	37038153	37038273
AMPL7170336175	chr3	37042398	37042517
AMPL7170087742	chr3	37042481	37042573
AMPL7170087112	chr3	37045873	37045962
AMPL7170335834	chr3	37048440	37048559
AMPL7170335741	chr3	37055857	37055975
AMPL7170094162	chr3	37056001	37056093
AMPL7170328392	chr3	37061833	37061961
AMPL7170325867	chr3	37067228	37067338
AMPL7158781874	chr3	37067336	37067464
AMPL7170329398	chr3	37067389	37067513
AMPL7171208508	chr3	37070287	37070413
AMPL7170329460	chr3	37081650	37081771
AMPL7170821030	chr3	37088962	37089085
AMPL7170336121	chr3	37090034	37090161
AMPL7170330529	chr3	37090414	37090539
AMPL7171894635	chr3	37091981	37092102
AMPL7170080659	chr3	41266008	41266088

AMPL7170365876	chr3	41266074	41266193
AMPL7170690627	chr3	41266215	41266334
AMPL7171902119	chr3	41266779	41266901
AMPL7170623698	chr3	41267179	41267299
AMPL7170352088	chr3	41268741	41268864
AMPL7170842474	chr3	41274850	41274974
AMPL7170157885	chr3	41275074	41275164
AMPL7170624092	chr3	41275721	41275846
AMPL7170352131	chr3	41277748	41277852
AMPL7170559141	chr3	41277840	41277948
AMPL7171404447	chr3	52437225	52437352
AMPL7170834031	chr3	52437830	52437956
AMPL7170181311	chr3	52438419	52438527
AMPL7170804220	chr3	52440199	52440325
AMPL7170372994	chr3	52441160	52441291
AMPL7171891937	chr3	52442007	52442102
AMPL7170804205	chr3	52442548	52442674
AMPL7170326525	chr3	142177739	142177861
AMPL7171902085	chr3	142177973	142178093
AMPL7170814928	chr3	142178119	142178236
AMPL7171032843	chr3	142180822	142180911
AMPL7171869174	chr3	142183953	142184068
AMPL7171891924	chr3	142185232	142185353
AMPL7171868947	chr3	142188259	142188379
AMPL7170326482	chr3	142215811	142215933
AMPL7171868863	chr3	142217511	142217632
AMPL7170326095	chr3	142238546	142238659
AMPL7170103762	chr3	142261500	142261573
AMPL7171901947	chr3	142269009	142269126
AMPL7171902055	chr3	142272644	142272729
AMPL7170326492	chr3	142274685	142274806
AMPL7170326041	chr3	142274794	142274915
AMPL7170104287	chr3	142274877	142274962
AMPL7171891854	chr3	142277513	142277604
AMPL7171622709	chr3	142281407	142281529
AMPL7171901920	chr3	142284920	142285039
AMPL7170318525	chr3	178916679	178916785
AMPL7171901951	chr3	178916742	178916866
AMPL7170318976	chr3	178916844	178916946
AMPL7170325752	chr3	178916935	178917035
AMPL7170318845	chr3	178917416	178917537
AMPL7170318269	chr3	178921449	178921571
AMPL7160402682	chr3	178927933	178928022
AMPL7170325785	chr3	178928046	178928160
AMPL7170365889	chr3	178936052	178936179
AMPL7170318556	chr3	178938829	178938960
AMPL7170081595	chr3	178947808	178947900
AMPL7170325662	chr3	178951990	178952114
AMPL7171901984	chr3	178952073	178952192
AMPL7170361219	chr4	1795640	1795780

AMPL7171196229	chr4	1795757	1795885
AMPL7170559504	chr4	1801357	1801492
AMPL7170361428	chr4	1801464	1801602
AMPL7170325769	chr4	1803463	1803603
AMPL7170359670	chr4	1805523	1805647
AMPL7170325644	chr4	1805961	1806096
AMPL7170325652	chr4	1806084	1806217
AMPL7171902000	chr4	1806450	1806570
AMPL7170158106	chr4	1807288	1807397
AMPL7170359823	chr4	1807700	1807838
AMPL7170157988	chr4	1807788	1807894
AMPL7171901927	chr4	1807882	1808016
AMPL7170102313	chr4	1808001	1808095
AMPL7171430650	chr4	1808212	1808347
AMPL7170623186	chr5	67522673	67522800
AMPL7171902039	chr5	67575383	67575502
AMPL7170577672	chr5	67589449	67589548
AMPL7171891942	chr5	67589553	67589659
AMPL7171891927	chr5	67589660	67589773
AMPL7171901942	chr5	67590980	67591100
AMPL7170577525	chr5	67591084	67591207
AMPL7170828919	chr5	67591221	67591329
AMPL7171902116	chr5	67591288	67591411
AMPL7170088270	chr5	67592016	67592122
AMPL7171033561	chr5	67592101	67592229
AMPL7170860761	chr5	67593212	67593338
AMPL7170373180	chr5	112102870	112102991
AMPL7171891920	chr5	112102914	112103030
AMPL7170372984	chr5	112111345	112111441
AMPL7170544588	chr5	112128084	112128207
AMPL7170548090	chr5	112128184	112128264
AMPL7170334386	chr5	112151205	112151324
AMPL7171869504	chr5	112154903	112155023
AMPL7171891857	chr5	112157564	112157670
AMPL7170545470	chr5	112157683	112157806
AMPL7170373328	chr5	112162799	112162920
AMPL7170544679	chr5	112170557	112170678
AMPL7171901940	chr5	112170690	112170814
AMPL7171901969	chr5	112170819	112170905
AMPL7171902014	chr5	112173335	112173432
AMPL7171901923	chr5	112173599	112173719
AMPL7171901980	chr5	112173787	112173909
AMPL7170372973	chr5	112173879	112174006
AMPL7170373507	chr5	112174215	112174331
AMPL7171777278	chr5	112174361	112174479
AMPL7171891892	chr5	112174506	112174579
AMPL7171323159	chr5	112174597	112174721
AMPL7164607197	chr5	112174815	112174941
AMPL7169188488	chr5	112175056	112175154
AMPL7171901999	chr5	112175143	112175225

AMPL7171901939	chr5	112175214	112175332
AMPL7157779721	chr5	112175462	112175586
AMPL7171901961	chr5	112175544	112175661
AMPL7170373235	chr5	112175647	112175739
AMPL7171902019	chr5	112175711	112175818
AMPL7171902050	chr5	112175799	112175909
AMPL7171902063	chr5	112175898	112176016
AMPL7170146404	chr5	112176003	112176083
AMPL7170373042	chr5	112176060	112176183
AMPL7170373409	chr5	112176162	112176286
AMPL7171902056	chr5	112176352	112176476
AMPL7170373503	chr5	112176492	112176607
AMPL7171902122	chr5	112176588	112176705
AMPL7171869496	chr5	112176720	112176835
AMPL7171902110	chr5	112176868	112176990
AMPL7171868765	chr5	112177056	112177180
AMPL7171868778	chr5	112177117	112177243
AMPL7171869438	chr5	112177301	112177414
AMPL7170373221	chr5	112177717	112177836
AMPL7170333377	chr5	112177927	112178046
AMPL7171868845	chr5	112178226	112178354
AMPL7157390263	chr5	112178783	112178907
AMPL7171902046	chr5	112178896	112179017
AMPL7160084437	chr5	112179330	112179454
AMPL7170331532	chr5	131895024	131895124
AMPL7171869422	chr5	131911539	131911659
AMPL7170331902	chr5	131924329	131924456
AMPL7171868901	chr5	131925265	131925374
AMPL7170336013	chr5	131931389	131931511
AMPL7170328702	chr5	131944334	131944455
AMPL7171868919	chr5	131953784	131953889
AMPL7170835541	chr6	35423706	35423832
AMPL7171366221	chr6	35428285	35428413
AMPL7170330552	chr7	6022596	6022721
AMPL7171894664	chr7	6026452	6026577
AMPL7170365694	chr7	6026887	6027021
AMPL7159075227	chr7	6027035	6027164
AMPL7171894628	chr7	6027115	6027248
AMPL7171902016	chr7	6029421	6029520
AMPL7170365803	chr7	6029522	6029638
AMPL7171828122	chr7	6035146	6035246
AMPL7171902080	chr7	6035209	6035322
AMPL7170331817	chr7	6036889	6036999
AMPL7164858338	chr7	6038698	6038825
AMPL7170333717	chr7	6038775	6038889
AMPL7170334660	chr7	6042131	6042266
AMPL7170088232	chr7	6042204	6042305
AMPL7170088986	chr7	6043281	6043374
AMPL7170333206	chr7	6043536	6043657
AMPL7170330659	chr7	6043609	6043724

AMPL7170330565	chr7	6045479	6045594
AMPL7170331042	chr7	6045600	6045727
AMPL7170118512	chr7	140434377	140434494
AMPL7170359467	chr7	140434532	140434653
AMPL7171902081	chr7	140439595	140439716
AMPL7170081580	chr7	140439717	140439791
AMPL7170360377	chr7	140449040	140449162
AMPL7170359652	chr7	140449187	140449289
AMPL7171208636	chr7	140453054	140453172
AMPL7170081632	chr7	140453141	140453240
AMPL7170359261	chr7	140453952	140454075
AMPL7171049748	chr7	140476653	140476773
AMPL7170944236	chr7	140476787	140476882
AMPL7170360403	chr7	140477785	140477882
AMPL7170422636	chr7	140477824	140477940
AMPL7170096617	chr7	140481364	140481460
AMPL7170359703	chr7	140481392	140481515
AMPL7170359932	chr7	140482905	140483003
AMPL7170420570	chr7	140494252	140494366
AMPL7170379969	chr7	140501259	140501377
AMPL7170421836	chr7	140534383	140534502
AMPL7170424778	chr8	26196460	26196565
AMPL7170424931	chr8	26220140	26220261
AMPL7171600342	chr8	26220318	26220444
AMPL7171869444	chr8	30915896	30916015
AMPL7170374195	chr8	30924654	30924777
AMPL7171869443	chr8	30938339	30938462
AMPL7171868871	chr8	30938585	30938704
AMPL7171869044	chr8	30969167	30969287
AMPL7170373885	chr8	30977842	30977963
AMPL7171049860	chr8	38271113	38271221
AMPL7170420507	chr8	38271597	38271723
AMPL7170360744	chr8	38272264	38272356
AMPL7171901973	chr8	38274782	38274885
AMPL7170252148	chr8	38274866	38274953
AMPL7171049679	chr8	38274930	38275056
AMPL7171404251	chr8	38276987	38277107
AMPL7171234762	chr8	38279311	38279432
AMPL7171049800	chr8	38281751	38281877
AMPL7170359931	chr8	38283615	38283730
AMPL7170201839	chr8	38285849	38285947
AMPL7170325799	chr8	38285870	38285999
AMPL7170359796	chr8	38297813	38297924
AMPL7170106044	chr8	90949243	90949321
AMPL7170813323	chr8	90955430	90955557
AMPL7170330164	chr8	90960022	90960142
AMPL7171891880	chr8	90965561	90965683
AMPL7171902108	chr8	90965708	90965828
AMPL7163020642	chr8	90965849	90965961
AMPL7171902002	chr8	90970975	90971094

AMPL7170332176	chr8	90982521	90982636
AMPL7170329541	chr8	90983313	90983431
AMPL7170365451	chr8	90983411	90983504
AMPL7171402333	chr8	90992919	90993030
AMPL7170107577	chr8	90993563	90993647
AMPL7170365302	chr8	90993622	90993738
AMPL7170331825	chr8	90994919	90995047
AMPL7160837073	chr9	21971084	21971223
AMPL7170330433	chr9	21974754	21974894
AMPL7170330820	chr9	21994137	21994253
AMPL7171869246	chr9	35075651	35075758
AMPL7162053769	chr9	35075907	35076029
AMPL7171891847	chr9	35076352	35076452
AMPL7170334025	chr9	97873754	97873866
AMPL7170331992	chr9	97887342	97887466
AMPL7170332179	chr9	97897682	97897801
AMPL7170332849	chr9	97934366	97934476
AMPL7171869333	chrX	14861776	14861901
AMPL7171869453	chrX	14862774	14862893
AMPL7169427003	chrX	14877351	14877463
AMPL7171902059	chrX	14882906	14883032
AMPL7171366357	chrX	14883225	14883346
AMPL7171869012	chrX	66764907	66765041
AMPL7171291690	chrX	66765458	66765586
AMPL7171902082	chrX	66765911	66766022
AMPL7170560676	chrX	66766432	66766565
AMPL7171725622	chrX	66863037	66863161
AMPL7170560663	chrX	66905815	66905939
AMPL7170559978	chrX	66931137	66931258
AMPL7170585068	chrX	66931238	66931358
AMPL7170560701	chrX	66931437	66931551
AMPL7170560393	chrX	66937284	66937413
AMPL7170560055	chrX	66937368	66937485
AMPL7171869508	chrX	66941670	66941791
AMPL7171308532	chrX	66941746	66941868
AMPL7170200880	chrX	66942612	66942712
AMPL7171403455	chrX	66942737	66942861
AMPL7170952694	chrX	66943456	66943560
AMPL7171032892	chrX	66943527	66943649
AMPL7170197272	chrX	76777844	76777941
AMPL7171891912	chrX	76812891	76813013
AMPL7171902096	chrX	76829758	76829879
AMPL7170423528	chrX	76845392	76845492
AMPL7170543898	chrX	76872120	76872237
AMPL7170543314	chrX	76889080	76889199
AMPL7170815021	chrX	76918742	76918852
AMPL7171868887	chrX	76918841	76918966
AMPL7170421029	chrX	76937571	76937688
AMPL7171032488	chrX	76937672	76937788
AMPL7171891894	chrX	76937990	76938117

AMPL7171225410	chrX	76938448	76938574
